# Trained immunity in newborn infants of HBV-infected mothers

**DOI:** 10.1038/ncomms7588

**Published:** 2015-03-25

**Authors:** Michelle Hong, Elena Sandalova, Diana Low, Adam J. Gehring, Stefania Fieni, Barbara Amadei, Simonetta Urbani, Yap-Seng Chong, Ernesto Guccione, Antonio Bertoletti

**Affiliations:** 1 Singapore Institute for Clinical Sciences, Agency for Science, Technology and Research (A*STAR), 30 Medical Drive, Singapore 117609, Singapore; 2 Emerging Infectious Diseases (EID) Program, Duke-NUS Graduate Medical School, 8 College Road, Singapore 169857, Singapore; 3 Institute of Molecular and Cell Biology, Agency for Science, Technology and Research (A*STAR), Singapore 138673, Singapore; 4 UOC Ostetricia e Ginecologia, Dipartimento Materno Infantile, Azienda Ospedaliero-Universitaria di Parma, Via Gramsci 14, 43126 Parma, Italy; 5 UO Immunoematologia e Medicina Trasfusionale, Dipartimento Diagnostico, Azienda Ospedaliero-Universitaria di Parma, Via Gramsci 14, 43126 Parma, Italy; 6 Department of Obstetrics and Gynaecology, Yong Loo Lin School of Medicine, National University of Singapore, National University Health System, 1E Kent Ridge Road, Singapore 119228, Singapore; 7 Department of Biochemistry, Yong Loo Lin School of Medicine, National University of Singapore, Block MD 7, 8 Medical Drive, Singapore 117597, Singapore; 8 School of Immunity and Infection, College of Medical and Dental Science, University of Birmingham, Edgbaston, Birmingham B15 2TT, UK

## Abstract

The newborn immune system is characterized by an impaired Th1-associated immune response. Hepatitis B virus (HBV) transmitted from infected mothers to newborns is thought to exploit the newborns’ immune system immaturity by inducing a state of immune tolerance that facilitates HBV persistence. Contrary to this hypothesis, we demonstrate here that HBV exposure *in utero* triggers a state of trained immunity, characterized by innate immune cell maturation and Th1 development, which in turn enhances the ability of cord blood immune cells to respond to bacterial infection *in vitro*. These training effects are associated with an alteration of the cytokine environment characterized by low IL-10 and, in most cases, high IL-12p40 and IFN-α2. Our data uncover a potentially symbiotic relationship between HBV and its natural host, and highlight the plasticity of the fetal immune system following viral exposure *in utero*.

## Abstract (web summary)

The ability to fight infections matures after birth and is thus termed ‘trained immunity’. Here the authors show that cord blood cells from hepatitis B virus-infected mothers respond more strongly to bacterial infections, suggesting that viral exposure *in utero* promotes trained immunity in newborns.

## Graphical abstract

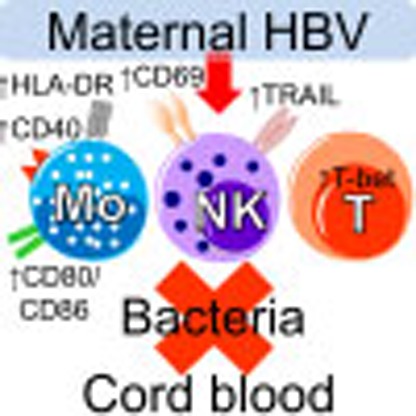

Infants have higher susceptibility to severe infections than adults due to functional differences in their immune system^1^,^2^. Hepatitis B virus (HBV) infection is a serious global health problem that causes liver inflammation and cancer in chronically infected adults^3^. As a large part of HBV chronic infections are acquired at birth, HBV is viewed as the prototypical pathogen that is thought to hijack the immaturity of the neonatal immune system and to establish a persistent infection through the induction of an ‘immunotolerant state’ in the host. Data from experimental animal models (that is, HBV-transgenic mice) showing the presence of immunological defects that impair T- and B-cell priming^4^,^5^,^6^ support this scenario.

However, the dogma of immune defects induced by HBV is at odds with the efficacy of HBV vaccination in infants born to HBV^+^ mothers^4^ and with observations obtained in malaria-HBV co-infected young subjects in whom reduced parasitemia^7^ and an increased incidence of cerebral malaria, a T-helper (Th)1-mediated malaria complication^8^,^9^, were reported. Recent data have also shown that chronically HBV-infected adolescents labelled as ‘immunotolerant’ do not display any tolerogenic T-cell features^10^.

In addition to these inconsistencies between experimental data in HBV models and data obtained during natural infection, there is an increased recognition that the neonatal immune system is not defective^11^. Instead, it presents unique functional features^11^,^12^,^13^ and the functional maturation of neonatal immunity can be modulated by external factors. For example, bacterial colonization and vaccinations with live vaccines can decrease infant mortality and protect them against unrelated pathogens by inducing an increased functional efficiency of their innate immune system that has been termed ‘trained immunity’^14^,^15^.

To directly characterize the impact of HBV exposure on the newborn immune system, we performed a comprehensive immunological analysis of the cord blood (CB) cells from neonates of HBV chronically infected mothers. We report that HBV exposure *in utero* does not induce generic immunological defects but, on the contrary, is associated with a mature immunological profile that enhances the ability of the neonatal immune cells to respond to unrelated pathogens *in vitro*.

## Results

### Cytokine profile in HBV-exposed CB

**Figure 1:**
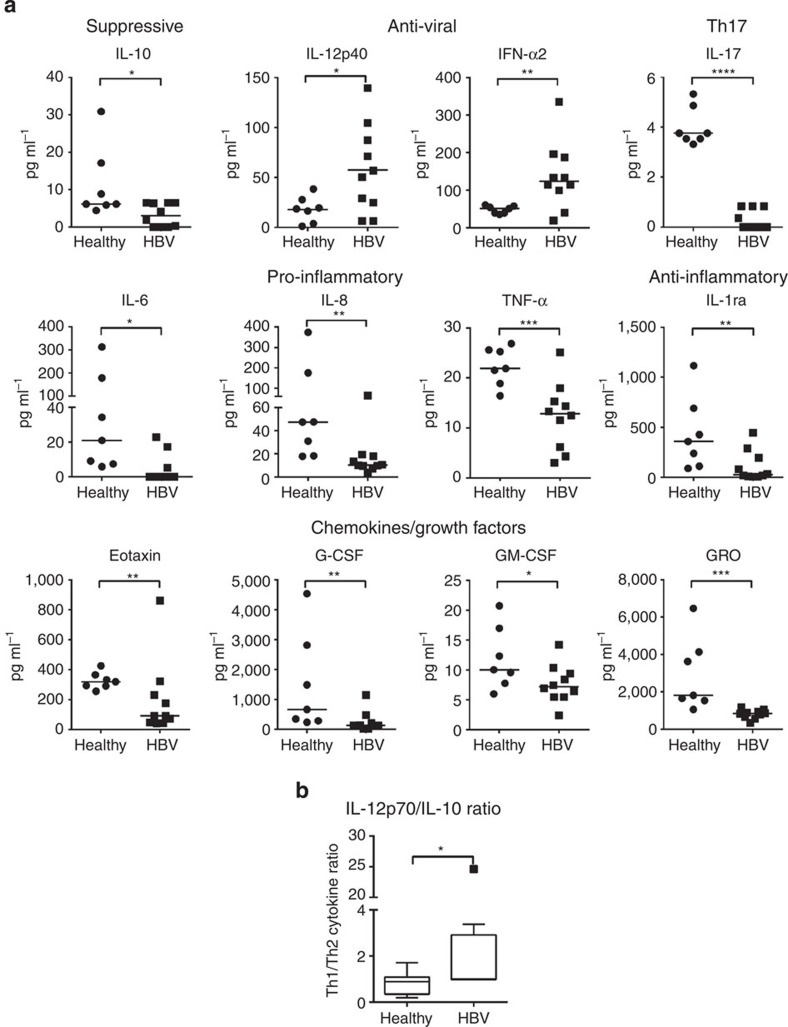
High levels of IL-12p40 and IFN-α2, and low levels of IL-10 and pro-inflammatory cytokines in the CB of Asian HBV^+^ mothers. (**a**) CB plasma cytokines were determined by multiplex assay in seven healthy controls and ten HBV^+^ mothers. Horizontal line represents the median. (**b**) Ratio of Th1/Th2 cytokine (IL-12p70/IL-10) in healthy and HBV-exposed CB. *P*-values were calculated using Mann–Whitney test. *P<0.05, **P<0.01, ***P<0.001 and ****P<0.0001.

We first analysed the cytokine profile of the umbilical CB plasma of HBV-exposed and healthy unexposed neonates of Asian origin (refer list of subject materials in Supplementary Table 1). The production of immunosuppressive cytokine (interleukin (IL)-10) was minimal (<10 pg ml^−1^) and significantly lower in the CB plasma of HBV^+^ mothers than controls (mean±s.e.m. in pg ml^−1^; healthy, 9.6±4.2, HBV, 1.3±0.7; Fig. 1a), whereas Th2 cytokines (IL-4, IL-5, IL-9 and IL-13) and pro-inflammatory cytokines (IL-1α and IL-1β) were undetectable in the CB plasma of HBV^+^ mothers and healthy controls (Supplementary Fig. 1). On the contrary, neonates born to HBV^+^ mothers had significantly higher plasma concentration of innate anti-viral cytokines IL-12p40 and interferon-α2a (IFN-α2) than healthy controls (mean±s.e.m. in pg ml^−1^; IL-12p40: 56.7±14.2 versus 18±5; IFN-α2: 137.3±28.1 versus 48.4±3.8; Fig. 1a). The presence of pro-inflammatory cytokines (IL-6, IL-8 and tumour necrosis factor-α (TNF-α)), anti-inflammatory cytokine (IL-1ra), Th17-related cytokine (IL-17) and neutrophil-related chemokines and growth factors (eotaxin, granulocyte-colony stimulating factor, granulocyte macrophage-colony stimulating factor and growth-regulated oncogene (GRO)) was instead significantly lower in the CB plasma of neonates of HBV^+^ mothers than controls (Fig. 1a). No difference was observed in the plasma levels of monocyte- and T-cell-attracting chemokines (MCP-1, MDC, MIP-1α, MIP-1β, Rantes; Supplementary Fig. 1). The elevated levels of IL-12p40 and IFN-α2 in the majority of the CB plasma in neonates born to HBV^+^ Asian mothers were unexpected, as the production of these cytokines are often low or undetectable both during acute HBV infection^16^ and during chronic HBV reactivation in adults^17^.

Importantly, we confirm this unique cytokine pattern (high IL-12p40 and low IL-10) in an independent cohort of CB samples from Caucasian HBV^+^ mothers (refer Supplementary Table 1 and Supplementary Fig. 2a,b). HBV-exposed CB plasma had increased IFN-α2 compared with healthy controls, although this difference was not statistically significant. The production of the pro-inflammatory cytokine IL-6 was minimal (<5 pg ml^−1^) and no difference was observed in the level of TNF-α. Nevertheless, HBV-exposed neonates of Caucasian HBV^+^ mothers showed decreased production of IL-8 in their CB plasma than controls, even though it was not statistically significant (Supplementary Fig. 2a). As type-III IFN was recently reported as an innate antiviral factor produced by human primary hepatocytes in response to HBV infection^18^, we tested the production of IFN-λ together with other type-I IFN (that is, IFN-β) in the CB plasma. Our data showed that although IFN-β was undetectable in all the CB plasma of both Asian and Caucasian cohorts, IFN-λ was only detectable in 1 out of 18 HBV-exposed CB plasma (Supplementary Figs 1 and 2a).

IL-12p40 has been reported to act either as a single cytokine or as a component of IL-23 or IL-12p70 (ref. 19), but we detected modest level of IL-12p70 (mean±s.e.m. in pg ml^−1^; healthy, 6.8±1.4, HBV, 6.5±0.1) and the absence of IL-23 in the CB plasma of neonates from HBV^+^ mothers (Supplementary Fig. 1), suggesting that IL-12p40 in the CB was present as a single agonistic cytokine^19^. Furthermore, we estimated the ratio of IL-12p70/IL-10 as an indication of a Th1/Th2 balance. Our data showed a significantly higher ratio of IL-12p70/IL-10 in HBV-exposed CB plasma than controls (mean±s.e.m. ratio; HBV, 4.3±2.9, healthy, 0.9±0.2) (Fig. 1b). This shift in Th1 cytokine balance was also observed in the independent cohort of CB samples from neonates of Caucasian HBV^+^ mothers (Supplementary Fig. 2b).

### HBV exposure *in utero* enhances innate immune activation

The detection of elevated levels of IL-12p40, combined with the detection of low IL-10 and Th2 cytokines, does not support the hypothesis that HBV induces a state of immune tolerance in newborns. Furthermore, elevated levels of IL-12p40 has been associated with sepsis control in newborns^20^, suggesting that this cytokine might be linked with increased immunological maturity. Therefore, we first analysed the frequency of different antigen-presenting cells (APCs) in HBV-exposed and healthy CB (Supplementary Fig. 3). The frequency of total APCs (or HLA-DR^+^ cells) and of the various APC subsets was not affected by HBV exposure *in utero*.

**Figure 2:**
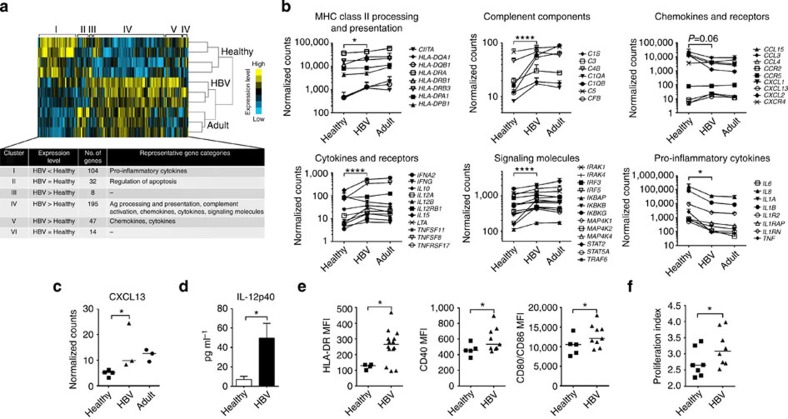
HBV exposure *in utero* enhances CB CD14^+^ monocyte maturation and activation. (**a**) Immune gene profiling on sorted CD14^+^ monocytes performed using Nanostring technology. Non-supervised hierarchical clustering of the expression of 400 immune-related genes differentially expressed between CD14^+^ monocytes from healthy (‘Healthy’, *n*=4) and HBV-exposed (‘HBV’, *n*=3) CB. CD14^+^ monocytes from healthy adult peripheral blood mononuclear cells (PBMCs; ‘Adult’, *n*=3) were included for comparison. Table shows the comparative mRNA levels, number of genes and representative gene categories in each gene cluster. (**b**) The differentially expressed genes between healthy and HBV-exposed CB monocytes can be broadly grouped into six gene categories. Graphs show the mean mRNA expression (in Nanostring counts) for representative immune genes in monocytes of healthy CB, HBV-exposed CB and adult peripheral blood within these six different gene categories. *P*-values between healthy and HBV-exposed CB were calculated using two-way analysis of variance with Bonferroni’s post test (refer to Supplementary Table 2). (**c**) The mRNA expression (in Nanostring counts) of the chemokine CXCL13 in monocytes measured with Nanostring technology. (**d**) Sorted CD14^+^ monocytes from healthy (*n*=4) and HBV-exposed (*n*=3) CB were incubated with 1 μg ml^−1^ ssRNA40 (TLR8 agonist) for 18 h and IL-12p40 in the supernatant measured using luminex. Data show mean±s.e.m. of each group. (**e**) The median fluorescence intensity (MFI) expression of HLA-DR, CD40 and CD80/CD86 on CD14^+^ monocytes from healthy (*n*=5) and HBV-exposed (*n*=10) CB. (**f**) Sorted CD14^+^ monocytes from healthy (*n*=7) or HBV-exposed (*n*=8) CB were incubated with allogeneic CFSE-labelled CD3^+^T cells (E:T ratio 1) for 7 days and CFSE staining analysed using flow cytometry. T-cell proliferation index was calculated using Flowjo. Horizontal line in dot plots represents the median. *P*-values in **c**–**f** were calculated using Mann–Whitney test, one-tailed. *P<0.05, ****P<0.0001.

In contrast, the functional profile of CD14^+^ monocytes, the most abundant population of innate immune cells present in the CB, is significantly different in HBV-exposed CB as compared with healthy controls. CD14^+^ monocytes were sorted directly *ex vivo* from the CB of healthy (*n*=4) or HBV^+^ Asian mothers (*n*=3; 2 HBeAg^−^, 1 HBeAg^+^; refer Supplementary Table 1), and were analysed for the expression of 511 immune genes with Nanostring technology^21^. There were no significant differences in immune gene profile between CB monocytes of HBeAg^+^ and HBeAg^−^ mothers, but notably a total of 400 immune genes were differentially expressed between HBV-exposed and healthy CB monocytes (Fig. 2a). Non-supervised hierarchical clustering showed six gene clusters corresponding to genes uniquely upregulated in healthy (cluster I, *n*=104) and HBV-exposed (cluster III, IV and V, *n*=8, *n*=195 and *n*=47, respectively) CB monocytes, as well as genes that were not distinctly enriched in either populations (cluster II and VI, *n*=32 and *n*=14, respectively) (Fig. 2a).

The differentially expressed genes between healthy and HBV-exposed CB monocytes can be broadly grouped into six different gene categories (Fig. 2b). Specifically, HBV-exposed CB monocytes expressed higher levels of messenger RNA associated with major histocompatibility complex class II processing and presentation, complement components, Th1-related cytokines (including IL-12p40 encoded by the mRNA *IL12B*, IFN-α2, IFN-γ and IL-15) and signalling molecules (Fig. 2b and Supplementary Table 2). Furthermore, the chemokine CXCL13, whose defect in HBV transgenic models has been recently suggested to predispose to HBV chronicity^6^, was significantly upregulated in HBV-exposed CB monocytes (Fig. 2c). On the other hand, the mRNA expression of pro-inflammatory cytokines (IL-1β, IL-6, IL-8 and TNF-α) was lower in HBV-exposed CB monocytes than healthy CB monocytes (Fig. 2b and Supplementary Table 2), further confirming the plasma cytokine data (Fig. 1a). Interestingly, the immune gene profile of HBV-exposed CB monocytes was more similar to the immune profile of healthy adult peripheral blood monocytes than that of the control CB monocytes, suggesting an increased immune maturation state of HBV-exposed CB monocytes (Fig. 2a,b). In addition, analysis of IFN-stimulated gene (ISG) expression revealed significant increase in the expression of several ISGs in HBV-exposed CB monocytes than controls (Supplementary Fig. 4), in line with the enhanced production of IFN-α2 in this cohort of HBV-exposed CB plasma (Fig. 1a).

The expression of some of these cytokines (that is, constitutively lower levels of pro-inflammatory cytokines IL-6, IL-8 and TNF-α, and the chemokines CCL3 and CCL4) was validated at the protein level in the supernatant of *ex vivo*-sorted monocytes (Supplementary Fig. 5). Direct *ex vivo* production of IL-12p40 or IFN-α2 was not detectable (Supplementary Fig. 5), but after activation with TLR8 agonist (ssRNA40)^13^ the production of IL-12p40 was markedly upregulated and was significantly higher in HBV-exposed CB monocytes than in controls (Fig. 2d).

*Ex vivo* phenotypic analysis confirmed the maturation and activation status of HBV-exposed CB monocytes. The levels of HLA-DR (HLA-class II presentation) and costimulation markers (CD40, CD80 and CD86) were significantly higher in HBV-exposed CB monocytes than in controls (Fig. 2e). Functionally, HBV-exposed CB monocytes induced a higher level of proliferation of allogeneic peripheral blood mononuclear cells than healthy CB monocytes (Fig. 2f).

In addition to monocytes, we have also analysed other components of innate immunity with anti-viral properties, including CD123^+^ plasmacytoid dendritic cells (pDCs) and natural killer (NK) cells (see Supplementary Table 1 for list of tested subjects). HBV-exposed CB pDCs were more activated than controls, characterized by significantly higher mRNA expression of several ISGs (Supplementary Fig. 6a) and higher production of IFN-α2 after stimulation with TLR9 agonist (CpG ODN2216; Supplementary Fig. 6b). There were no significant differences in the frequencies of NK subsets between healthy and HBV-exposed CB (Supplementary Fig. 7a). However, HBV-exposed CB NK cells displayed a more activated profile, as shown by increased frequencies and expression of TNF-related apoptosis-inducing ligand (mean±s.e.m. in percentages; CD56^br^: healthy 4.7±1.5, HBV 16.9±5.6; CD56^dim^: healthy 0.2±0.1, HBV 0.8±0.3) and the activation marker CD69 (mean±s.e.m. in percentages; CD56^dim^: healthy 13.5±1, HBV 18.1±1.2). HBV-exposed CB NK cells also had increased production of IFN-γ after incubation with recombinant IL-12p70 and IL-18 compared with healthy controls (mean±s.e.m. in pg ml^−1^; healthy 651.5±414.8, HBV 3,477±1,464) (Supplementary Fig. 7b–d).

### HBV exposure *in utero* induces robust Th1-polarized response

Newborn T cells produce IL-8 but are defective in Th1 cytokine production^11^. As IL-12p40 has been shown to increase IFN-γ production in adult T cells, we analysed the ability of CB T cells to produce Th1 and other important T-cell cytokines (that is, IL-17, IL-21 and IL-22).

**Figure 3:**
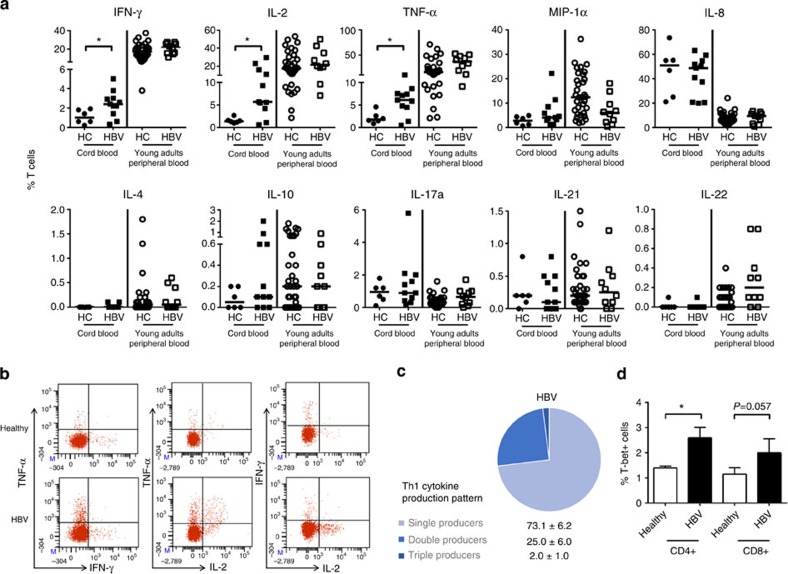
HBV exposure *in utero* induces a robust Th1-polarized response in the CB. (**a**) CB mononuclear cells were stimulated overnight with phorbol myristate acetate (PMA)/ionomycin and the cytokine production by CD3^+^T cells was measured using intracellular cytokine staining. Dot plots show the percentages of cytokine-producing CD3^+^T cells from healthy (HC; *n*=6) or HBV-exposed (HBV; *n*=11) CB. Cytokine production by CD3^+^T cells from the peripheral blood of pediatric and young adult patients with chronic HBV (HBV; *n*=10) and age-matched healthy controls (HC; *n*=33) were included for comparison. Horizontal line represents the median. (**b**) Representative FACS dot plots of Th1 cytokine (TNF-α, IFN-γ and IL-2) production from healthy and HBV-exposed CB T cells after PMA/ionomycin stimulation. (**c**) Graphical representation of single-, double- and triple-producer Th1 cells and their respective percentages in mean±s.e.m. in HBV-exposed CB. (**d**) Percentage of T cells expressing the Th1 marker, T-bet, in CB of healthy (*n*=4) and HBV^+^ (*n*=4) mothers. *P*-values were calculated using Mann–Whitney test.*P<0.05.

Figure 3a shows the frequency of CB CD3^+^T cells producing the indicated cytokines after polyclonal stimulation, in comparison with CD3^+^T cells present in healthy or HBV-infected young adults (12–30 years). As expected, both HBV-exposed and healthy CB T cells produced higher levels IL-8 but lower levels of IFN-γ, IL-2 and TNF-α, compared with young adults’ T cells. The ability to produce IL-8 was similar in HBV-exposed CB T cells compared with controls, while a significantly higher frequency of T cells producing Th1 cytokines was detected in HBV-exposed CB (mean±s.e.m. in percentages; IFN-γ: 2.4±0.4 versus 1.1±0.3; IL-2: 10.2±2.8 versus 1.6±0.2; TNF-α: 5.8±0.9 versus 2.2±0.5). A representative fluorescence-activated cell sorting (FACS) dot plot of Th1 cytokine production by CB T cells is shown in Fig. 3b. Analysis of the Th1 (IFN-γ, IL-2 and TNF-α) double- and triple-producer T cells showed that ~25% of HBV-exposed CB Th1 T cells were polyfunctional (mean±s.e.m. in percentages; single: 73.1±6.2, double: 25±6, triple: 2±1; Fig. 3c).

The increased Th1 maturation in HBV-exposed CB was confirmed by direct *ex vivo* analysis of T cells expressing T-bet, the transcriptional regulator of Th1 differentiation (Fig. 3d). No differences were found between CB T cells of HBV-exposed or healthy controls in their ability to produce IL-17, IL-22 and IL-21 (Fig. 3a), even though decreased IL-21 production has been implicated in HBV vertical infection and chronicity^22^.

Despite having a more Th1-polarized response, HBV-exposed neonates do not seem to harbour any HBV-specific T cells. Various attempts to detect HBV-specific T cells in HBV-exposed CB were unsuccessful. CB cells were analysed directly *ex vivo* with HBV-specific HLA tetramers or after *in vitro* expansion with peptides covering the whole HBV proteome. However, we were not able to detect any clear population of HBV-specific T cells in the CB of HBV-exposed neonates (Supplementary Fig. 8).

### HBV exposure *in utero* triggers a state of trained immunity

We next analysed whether the enhanced immune maturation detected in HBV-exposed CB could result in a better ability of the neonatal immune cells to respond to unrelated pathogens.

**Figure 4:**
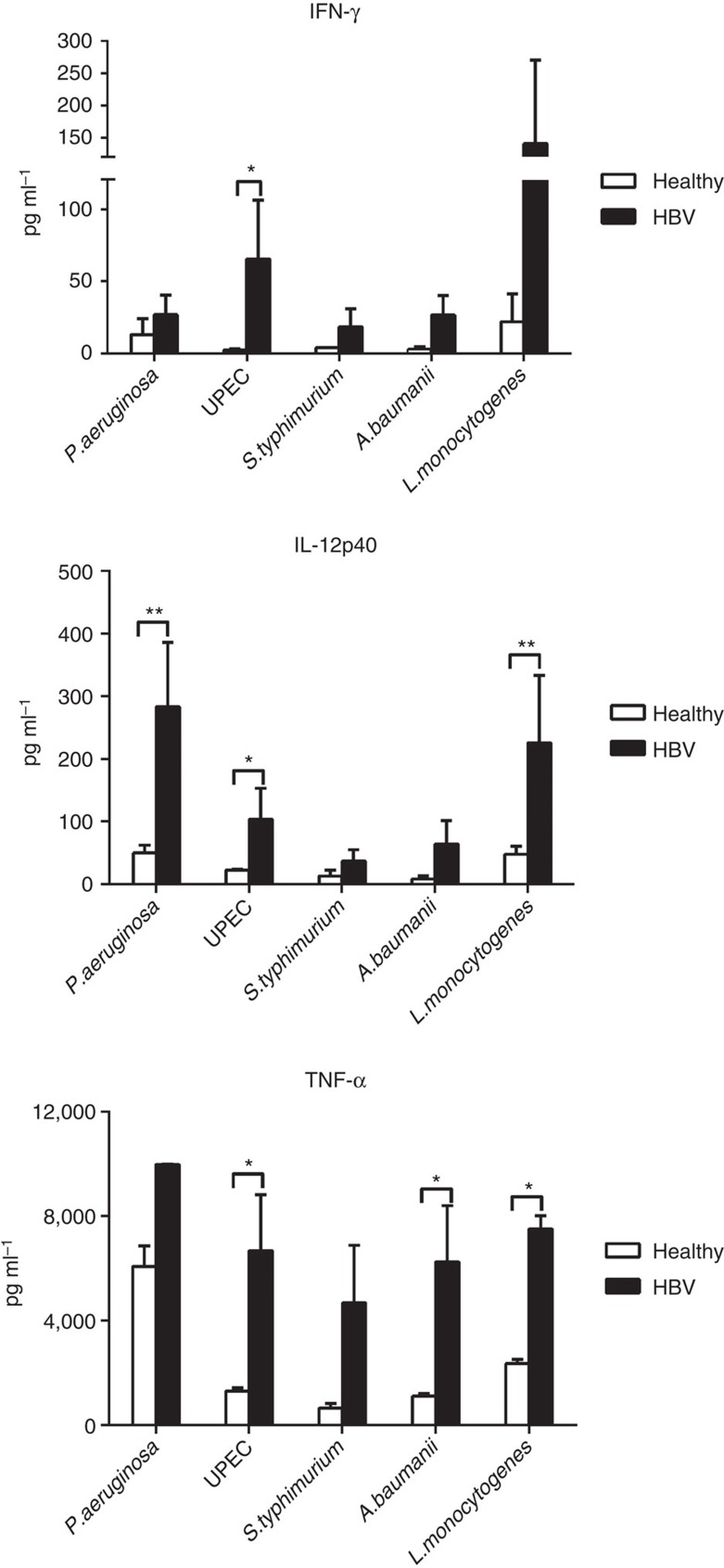
HBV exposure *in utero* triggers a state of ‘trained immunity’ against unrelated bacterial challenge. CB mononuclear cells from healthy (*n*=3) or HBV^+^ (*n*=3) mothers were incubated with the bacteria *P. aeruginosa*, *UPEC*, *S. typhimurium*, *A. baumanii* or *L. monocytogenes* (multiplicity of infection (MOI) 1) for 18 h and the cytokine production in the supernatant was analysed using multiplex assay. Bar graphs show the mean±s.e.m. of each data set. *P*-values were calculated using two-way analysis of variance and multiple comparisons were done using uncorrected Fisher’s least significant difference test. *P<0.05 and **P<0.01.

We tested CB mononuclear cells against *Pseudomonas aeruginosa*, a bacteria that can cause severe infections in underweight neonates^23^, as well as other bacteria known to be involved in neonatal sepsis in the clinics, such as uropathogenic *Escherichia coli* (*UPEC*), *Salmonella typhimurium*, *Acinetobacter baumanii* and *Listeria monocytogenes*. Cytokine production in the supernatant was measured after 18 h of bacteria stimulation and we detected a strong Th1 cytokine signature (IFN-γ, IL-12p40 and TNF-α) in bacterial-stimulated HBV-exposed CB compared with healthy controls (Fig. 4). Specifically, the production of IFN-γ was increased significantly when HBV-exposed CB cells were challenged with *UPEC*, IL-12p40 production was significantly higher after exposure to *P. aeruginosa*, *UPEC* and *L. monocytogenes*, and TNF-α production was significantly elevated on exposure to *UPEC*, *A. baumanii* and *L. monocytogenes*, compared with controls. Similar trend for higher production of Th1 cytokines was observed after exposure to *S. typhimurium*. Therefore, our data demonstrates that HBV exposure *in utero* increased the nonspecific production of Th1 cytokines towards unrelated pathogen challenge *in vitro*.

### HBV-induced immunological changes are neonatal in origin

**Figure 5:**
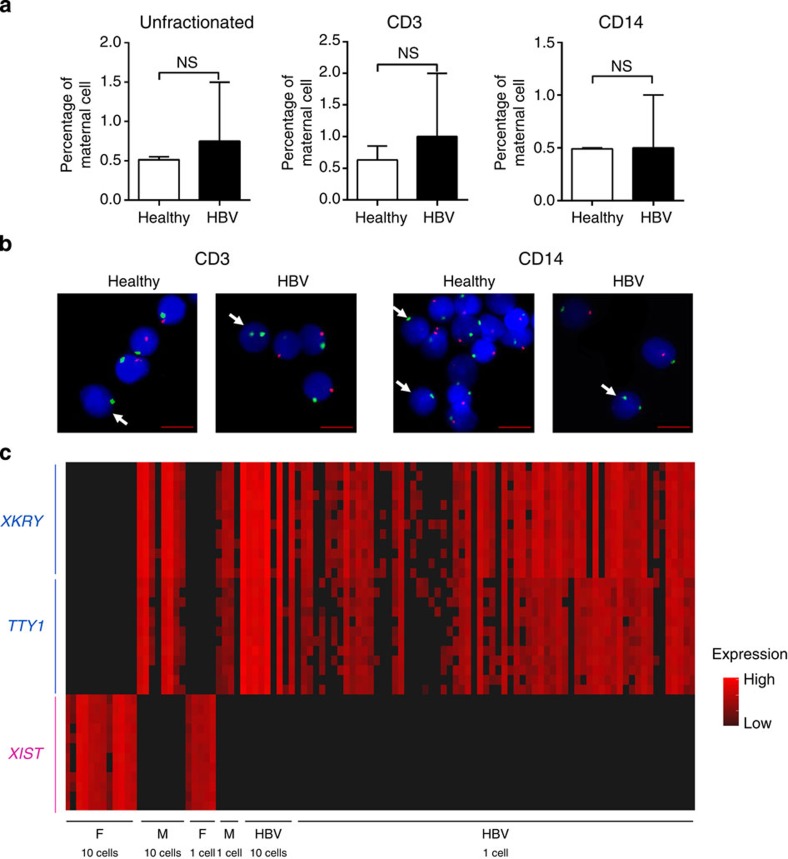
HBV-induced immune cell maturation is neonatal in origin. (**a**) FISH analysis with X- and Y-chromosome-specific probes to detect maternal cells in male CB. Graphs show the percentages of maternal cells (mean±s.e.m.) in CB unfractionated and fractionated cells (*n*=2 per group). Statistical significance between groups was calculated using Mann–Whitney test, one-tailed. *P*-value <0.05 is considered statistically significant. NS denotes nonsignificant. (**b**) Representative FISH images from each sorted cell population in healthy and HBV-exposed CB. Nuclei were counterstained with DAPI (blue), X- and Y-chromosomes in green and red, respectively. Maternal cells (white arrow) contain green chromosome X signals and no Y signals. Scale bar, 5 μm. (**c**) Quantitative PCR analysis using 48:48 Dynamic Arrays (Biomark System-Fluidigm) of CD14^+^ single cells derived from HBV-exposed CB of male neonate. Two male (M)-specific genes (*XKRY* and *TTY1*) are expressed in CB cells and not in female (F) cells. Similarly, *XIST* gene is selectively expressed in female cells. Black, no detected expression.

The increased immune maturation state detected in HBV-exposed CB cells could be due to either functional changes of neonatal monocytes/T cells or increased frequency of maternal immune cells in HBV-exposed CB. Immune cells of the mother are known to cross the placenta^24^, but whether HBV infection might have an effect on the degree of CB micro-chimerism is not known. We therefore quantified the frequency of maternal cells in HBV-exposed CB using two alternative methods. We first used fluorescence *in situ* hybridization (FISH) to quantify the number of maternal cells (expressing XX chromosomes) among bulk cells or sorted cells (CD14^+^ monocytes or CD3^+^T cells) from healthy and HBV-exposed CB of male neonates (*n*=2 per group). No significant differences in the frequencies of maternal cells between HBV-exposed and healthy CB were detected (Fig. 5a,b). The mean frequencies of maternal cells in healthy and HBV-exposed CB, respectively, were 0.52% versus 0.75% (bulk), 0.64% versus 1% (CD3^+^T cells) and 0.49% versus 0.5% (CD14^+^ monocytes; Fig. 5a).

We then used quantitative PCR on single cells to measure the expression of genes selectively expressed only by maternal (female) cells (*XIST* long noncoding RNA)^25^ or male neonates (*XKRY* and *TTY1* genes)^26^ as an alternative method to confirm that HBV-exposed CB was not preferentially enriched with maternal cells. A total of 136 CD14^+^ cells from one HBV-exposed CB male neonate were analysed and we did not detect any maternal cells (0/136; Fig. 5c). This gave us a frequency of maternal cell in CD14^+^ monocytes of HBV-exposed CB to be <0.7%, which is in line with the data obtained with FISH analysis (Fig. 5a) and with the reported frequency of maternal cells in healthy CB^27^. These results demonstrate that the immunological changes observed in HBV-exposed CB is unlikely to be due to an increase in maternal cell contamination, but probably due to genuine maturation of the neonatal immune cells.

### HBV-induced immune maturation is associated with HBsAg

Conventionally, prenatal HBV infection is thought to occur in a minority of cases, as HBV-DNA, a sign of active HBV replication, is only detected in the CB of a few HBeAg^+^ mothers^28^. On the other hand, HBV can translocate efficiently through intact trophoblastic barrier at early gestation^29^, but as the liver develops only after 12 weeks of gestation^30^ HBV infection and replication in hepatocytes might not occur.

To better understand the mechanisms responsible for the functional maturation of the immune cells present in the CB of neonates born to HBV^+^ mothers, we tested whether HBV or HBV products can be traced in their CB plasma or mononuclear cells. HBV-DNA was detected in only two of the four CB plasma of neonates born to HBeAg^+^ mothers (Supplementary Table 1) and was undetected in all the other CB plasma of HBeAg^−^ mothers. In addition, we were unable to detect the presence of HBV-DNA in the CB mononuclear cells from healthy (*n*=2), HBeAg^−^ (*n*=2) and HBeAg^+^ (*n*=1) mothers (Supplementary Table 1), despite the latter being tested positive for HBV-DNA in the CB plasma.

**Figure 6:**
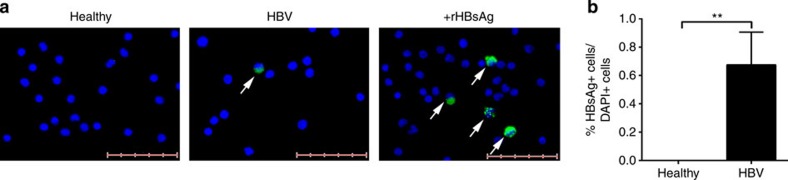
HBV-induced immune cell maturation is associated with the presence of HBsAg. (**a**) CD2^−^ cells were enriched from CB using MACS beads and the presence of HBsAg was detected using immunofluorescent staining. Detection of HBsAg in CB cells (white arrow) from healthy and HBV^+^ mothers (green–HBsAg; blue–DAPI). Images are representative of seven CB samples per group. CB cells incubated with recombinant HBsAg was used as positive control. Scale bar, 50 μm. (**b**) Percentage of HBsAg^+^ cells among DAPI^+^ cells in healthy and HBV-exposed CB (*n*=7 per group). *P*-value was calculated using Mann–Whitney test. **P<0.01.

However, as in woodchuck HBV model the woodchuck HBV can persist selectively in the blood of offspring of animals with very low level of viral replication^31^, we used immunofluorescence^32^ to test whether HBV or HBV products can be found in populations of purified CB immune cells. We purified CB CD2^−^ cells (enriched for APCs), stained them for hepatitis B surface antigen (HBsAg) and the number of HBsAg^+^ cells was then quantified in ten random fields at × 20 magnification (Fig. 6a). Despite most of the neonatal plasma of HBV^+^ mothers were negative for HBV-DNA, HBsAg^+^ cells were detectable in CD2^−^ cells (APC-enriched cells) but not in CD2^+^ (T and NK) cells at a mean frequency of 0.6±0.2% (Fig. 6b). No positive immunostaining for HBsAg was detected in healthy CB (Fig. 6a,b).

Thus, despite their low quantity, the presence of HBsAg^+^ cells in the CB of neonates of HBV^+^ mothers indicates that their immune system has been in contact with the virus or viral products before birth.

### IL-12p40 and IFN-α2 induce CB immune cell maturation

The low frequency of HBsAg^+^ immune cells detected in the CB of HBV-exposed neonates suggests that it is unlikely that HBV antigens can directly cause the maturation of monocytes in HBV-exposed CB. We hypothesize that the altered cytokine environment detected in the CB plasma of HBV-exposed neonates could be responsible for the induction of monocyte/T-cell maturation.

**Figure 7:**
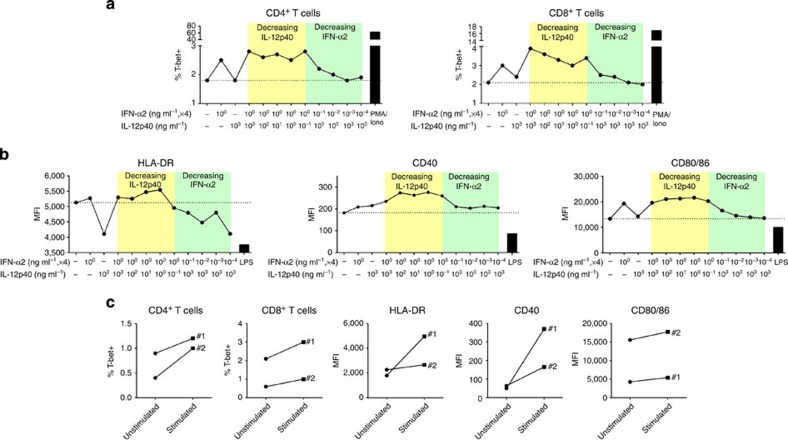
IL-12p40 and IFN-α2 trigger the maturation of healthy CB immune cells *in vitro*. CB mononuclear cells from healthy mothers (*n*=3) were incubated with rhIL-12p40 or rhIFN-α2, either alone or in different combinations of concentrations, overnight and the activation phenotypes of T cells and monocytes were analysed by FACS. Graphs (**a**,**b**) show the data from one representative sample. Dotted lines indicate the basal level without any cytokine stimulation. (**a**) The percentage of T-bet^+^ cells in CD4^+^ and CD8^+^ CB T cells. (**b**) The median fluorescence intensity (MFI) of expression of HLA-DR, CD40 and CD80/CD86 on CB monocytes. (**c**) The activation phenotypes of T cells and monocytes in CB cells (*n*=2, denoted 1 and 2) stimulated with physiological concentrations of rhIL-12p40 (0.1 ng ml^−1^) and rhIFN-α2 (0.4 ng ml^−1^).

Thus, to mimic the altered cytokine environment, we incubated CB cells from healthy mothers (*n*=3) overnight with different concentrations of recombinant human (rh) IFN-α2 (0.0004–4 ng ml^−1^) and rhIL-12p40 (0.1–1,000 ng ml^−1^), alone or in combination, and analysed the activation of T cells and monocytes by flow cytometry. The data for one representative CB sample is shown in Fig. 7a,b. Specifically, rhIFN-α2 alone (4 ng ml^−1^) was able to promote Th1 development and monocyte maturation of healthy CB cells compared with no cytokine controls, while high dose of rhIL-12p40 alone (1,000 ng ml^−1^) had modest effects. However, a combination of both rhIFN-α2 (4 ng ml^−1^) and rhIL-12p40 (1,000 ng ml^−1^) further increased the frequency of T-bet expressing CD4^+^ and CD8^+^ T cells than either rhIFN-α2 or rIL-12p40 alone. At lower doses of rhIL-12p40 (1–10 ng ml^−1^), in combination with high dose of rhIFN-α2 (4 ng ml^−1^), the median fluorescence intensity of HLA-DR, CD40 and CD80/CD86 on CB monocytes was upregulated compared with using both cytokines at high doses (mean median fluorescence intensity; 1 ng ml^−1^ rhIL-12p40; HLA-DR, 5,542 versus 5,294; CD40, 276 versus 235; CD80/CD86, 21,623 versus 19,571). There was a clear dose effect for rhIFN-α2 in Th1 development (with fixed concentration of rhIL-12p40 at 1,000 ng ml^−1^) and this effect was abolished at 0.004 ng ml^−1^ of rhIFN-α2. For rhIL-12p40, there was a trend for increased monocyte activation with decreasing rhIL-12p40 (with fixed concentration of rhIFN-α2 at 4 ng ml^−1^) and this effect peaked at ~1 ng ml^−1^ of rhIL-12p40. Most importantly, the increase in T-bet^+^ T cells and the enhanced activation of monocytes were observed when CB cells (*n*=2) were incubated with rhIFN-α2 and rhIL-12p40 at concentrations observed in the HBV-exposed CB plasma (rhIFN-α2–0.4 ng ml^−1^ and rhIL-12p40–0.1 ng ml^−1^; Fig. 7c).

## Discussion

The concept of trained innate immune responses has been documented in plants^33^,^34^, invertebrates^35^,^36^,^37^, mice^38^,^39^,^40^ and, more recently, in vaccinated humans^41^. However, no such evidence has been demonstrated so far in newborns during the course of a natural viral infection. In this work, we demonstrated that HBV exposure *in utero* induces a state of trained immunity characterized by enhanced innate immune cell maturation and increased Th1 development. Importantly, this immune system maturation results in a better ability of the neonatal immune cells to respond to unrelated pathogen exposure.

Additional immunological changes observed in HBV-exposed neonates were the higher production of IL-12p40 and lower production of IL-10 and pro-inflammatory cytokines (IL-6, IL-8 and TNF-α) in the CB plasma than controls. This immunological pattern (high Th1-related/low IL-10 and pro-inflammatory cytokines) was also observed in HBV-exposed CB monocytes. Therefore, HBV exposure *in utero* induced complex changes in the newborn’s immune system that were not exclusively stimulatory in nature but were generally compatible with an advanced immune maturation state. Indeed, during the first year of life^12^, the infant’s immune system does not only acquire a more pronounced Th1 T-cell profile but also decrease its ability to produce IL-10 and pro-inflammatory cytokines.

A further alteration induced by HBV exposure was the detection of higher levels of IFN-α2 that were only statistically significant in the CB plasma of Asian but not Caucasian HBV^+^ mothers. Whether such differences could be explained by different HBV genotypes infecting the two cohorts (HBV genotypes B/C in Asian patients versus HBV genotype D in Caucasian patients) will require further analysis.

Epidemiological, clinical and experimental evidences have already raised doubts about the concept of immunological tolerance during HBV infection^42^. Indeed, our data show that HBV, a virus thought to exploit the immaturity of the neonatal immune system to establish chronic infection, was unexpectedly inducing a state of ‘trained immunity’ with a more pronounced Th1 profile. However, in contrast to the evidences of global immune system maturation, we were not able to detect any HBV-specific T-cell response in the CB of HBV-exposed neonates. This is in contrast to the data obtained in human cytomegalovirus^43^ and human immunodeficiency virus infections^44^, where virus-specific T cells can be detected in neonates. A possible scenario is that HBV has evolved a special relationship with its human host: although the defective priming of HBV-specific T cells can predispose to HBV chronicity, the induction of a trained immunity profile with a skewed Th1 response and suppression of pro-inflammatory events might have the advantage of decreasing mortality from exposure to unrelated pathogens.

Nonetheless, more data need to be gathered to fully understand the impact of vertical HBV infection on its host. A limitation of our study is that as HBV-exposed neonates must be vaccinated and treated within 24 h of birth, we were unable to investigate the consequences of the establishment of a persistent HBV infection after birth. It could be possible that the establishment of chronic HBV infection in neonates may be associated with a more robust and persistent Th1 response and a better ability to control unrelated pathogens, or it may be associated with defects in the priming of adaptive immunity, as shown in HBV-transgenic mice^6^. We posit that the evidences of trained immunity shown here are more in line with our recent demonstration that young CHB-infected patients present a fully normal Th1 T-cell profile and do not show any increased defects in HBV-specific T-cell repertoire compared with HBV-infected adults^10^, but certainly a more precise evaluation of the immunological events that are occurring in the early phases of HBV infection is needed.

The question of how HBV exposure is inducing trained immunity is still open. The low frequency of HBsAg^+^ monocytes detected in the CB of HBV-exposed neonates does not support the scenario of a direct HBV infection of the neonates triggering trained immunity. It is perhaps more likely to be that the cause of induction of trained immunity lies with the cytokine environment detected in the HBV-exposed newborns, characterized by an increase production of IL-12p40 and, at least in some cases, IFN-α2. Both IL-12p40 and IFN-α2 have been shown to skew T-cell development towards Th1 maturation^19^,^45^,^46^,^47^, and incubation of CB cells with these recombinant cytokines in our *in vitro* study supports such a possibility. Epigenetic-mediated functional reprogramming of immune cells has been reported as one of the mechanisms mediating trained immunity^41^, but whether such epigenetic events are occurring in HBV-exposed neonates requires further investigations. Certainly, our *in vitro* assays are a profound approximation of the events that are occurring during the natural development of the newborn immune system and a clear answer to this question will probably require the use of the woodchuck hepatitis B animal model wherein viral transmission from mother to offspring can occur even in the presence of extremely low quantity of virus^31^.

The source of IL-12p40, the cytokine that was found to be consistently elevated in the CB plasma of HBV-exposed neonates, is also at the moment unknown. *Ex vivo* production of IL-12p40 in sorted monocytes from HBV-exposed CB was not detectable, suggesting that despite the natural contact with HBV products, the circulating monocytes were not directly responsible for this cytokine production. A possibility is that the high levels of IL-12p40 detected in the HBV-exposed CB may be produced by other types of haematopoietic phagocytic cells, such as myeloid DCs or neutrophils. Alternatively, the placenta, which consists of multiple layers of cell barriers with precise immune function and has been shown to harbour HBV and HBV products^48^, might actually be the source of IL-12p40 production in response to HBV. Pregnancy is known to modulate the natural history of HBV infection, but whether placental cells can actually play a direct role in the modulation of maternal or neonatal infections remains unknown.

Despite these limitations, our data clearly show a novel interaction between HBV and its human host. The evidences of immune system maturation in the newborns as a result of HBV exposure *in utero* suggests the presence of a symbiotic relationship between HBV and humans, similar to that demonstrated in mice with persistent herpes simplex virus infection^49^. This symbiotic host–virus interaction could be the explanation as to why HBV has been so efficient in co-existing in a large part of the human population from its dawn^50^.

## Methods

### Patients and blood samples

Umbilical CB was obtained from two independent cohort of patients: the first cohort consists of 20 neonates born to HBV-seropositive women (HBV-exposed group) and 7 neonates born to HBV-seronegative women (non-exposed control group). The deliveries occurred at the National University Hospital, Singapore, and all women were of Asian ethnicity (Chinese or Malay). The second cohort consists of eight neonates born to HBV^+^ women and four neonates born to HBV^−^ women. The deliveries occurred at the Dipartimento Materno Infantile, Azienda Ospedaliero Universitaria di Parma, Italy, and the women were of Caucasian ethnicity. None of the HBV-infected mothers in both cohorts received antiviral treatment before delivery. Basic clinical and demographic data were collected at the time of delivery (Supplementary Table 1). Maternal serum was tested for HBsAg, HBeAg and HBV DNA level (a few patients). All mothers in the HBV group were positive for HBsAg and negative for human immunodeficiency virus. At delivery, CB was collected from the umbilical vein using a direct dripping method into tubes containing heparin. Subsequently, plasma was separated from whole blood and stored at −20 °C and CB mononuclear cells were isolated by density-gradient centrifugation on Ficoll-Hypaque. The study in Singapore was approved by the Domain Specific Review Board at National University Hospital, which was in accordance with the guidelines of the Singapore National Healthcare Group. The study in Italy was approved by the Comitato Etico Azienda Ospedaliero Parma (Protocol 6274) and was in accordance with the guidelines of the Italian Minister of Health. Blood samples from 10 pediatric and young adult CHB patients (12–30 years old) and 33 age-matched healthy controls used for the Th1 T-cell analysis were obtained from a viral hepatitis clinic at The Royal London Hospital, UK. Ethics approval was obtained from Barts and The London NHS Trust Ethics Review Board. All donors gave written informed consent.

### Antibodies and reagents

Monoclonal anibodies (mAbs) of anti-human-CD3-eFluor 605NC (clone OKT3, 3:100), anti-CD4-eFluor 650NC (RPA-T4, 3:100), anti-CD7-FITC (4H9, 2.5:100), anti-CD11c eFluor 450 (3.9, 1.25:100), anti-HLA-DR-Alexa Fluor 700 (LN3, 5:100) or –eFluor 605NC (LN3, 2:100) and anti-T-bet (eBio4B10, 1:100) were obtained from eBioscience (San Diego, CA). Anti-CD3-FITC (HIT3a, 2.5:100), anti-CD11b-PE-Cy7 (ICRF44, 5:100), anti-CD16-APC-Cy7 (3G8, 2.5:100) or –BV711 (3G8, 5:100), anti-CD19-FITC (HIB19, 2.5:100), anti-CD20-FITC (2H7, 2.5:100), anti-CD56-FITC (HCD56, 2.5:100), anti-CD86-APC (IT2.2, 6:100) and anti-CD123-PerCP-Cy5.5 (6H6, 2.5:100) were obtained from Biolegend (San Diego, CA). Anti-CD3-PE-Cy7 (SK7, 2:100), anti-CD8-V500 (RPA-T8, 1:100), anti-CD11c-V450 (B-Ly6, 1.25:100), anti-CD14-PE-Cy7 (M5E2, 2.5:100), anti-IL17a-V450 (N49-653, 4:100), anti-CD40-Alexa Fluor 700 (5C3, 6:100), anti-CD45-V500 (HI30, 2:100), anti-CD56-V450 (B159, 2:100), anti-CD80-PE (L307.4, 6:100), anti-IFNγ-V450 (B27, 6:100) or -PerCP-Cy5.5 (B27, 6:100), anti-IL-2-PerCP-Cy5.5 (MQ1-17H12, 4:100), anti-IL-4-FITC (MP4-25D2, 4:100), anti-IL-8-PE (G265-8, 2:100), anti-IL-10-APC (JES3-19F1, 6:100), anti-IL-21-PE (3A3-N2.1, 6:100), anti-MIP-1β-PE-Cy7 (D21-1351, 4:100), anti-TNF-related apoptosis-inducing ligand-PE (RIK-2, 6:100) and anti-TNF-α-PE-Cy7 (MAb11, 6:100) were obtained from Becton Dickinson (BD, San Jose, CA). mAbs of anti-IL-22-APC (142928, 10:100) and anti-MIP-1α-FITC (93342, 4:100) were obtained from R&D Systems (Minneapolis, MN). mAbs of anti-CD3-eFluor 605NC (UCHT1, 6:100) was obtained from Molecular Probes (Carlsbad, CA). mAb of anti-CD14-ECD (RMO52, 2.5:100) was obtained from Beckman Coulter (Brea, CA). Live/Dead Fixable Dead Cell Stain Kits (yellow and aqua, 1:1,000 in 1 × PBS) were obtained from Invitrogen. Agonists for human TLR4 (*E. coli* K12 LPS, 1 μg ml^−1^), TLR8 (ssRNA40, 1 μg ml^−1^) and TLR9 (CpG ODN2216, 5 μM) were obtained from Invivogen (San Diego, CA). Phorbol myristate acetate (2 ng ml^−1^) and ionomycin (1 μg ml^−1^) were obtained from Sigma-Aldrich (Saint Louis, Missouri).

### HBV DNA detection

HBV DNA was isolated from CB plasma or CB mononuclear cell lysates using the High Pure Viral Nucleic Acid Kit (Roche Applied Science). For the CB mononuclear cells, the cells were lysed in RLT buffer (Qiagen) and the lysate was passed through a blunt 20-gauge needle (0.9 mm diameter) fitted to an RNase-free syringe. The lysate was centrifuged and the supernatant collected for HBV DNA extraction. Five microlitres of internal control (HBV RG/TM IC) from the Artus HBV RG PCR kit (Qiagen) was added to the mixture of sample material and lysis buffer to control for the purification process. HBV DNA was quantified using the Artus HBV RG PCR kit (Qiagen) on a Rotor-Gene Q platform, according the manufacturer’s protocol. The 95% HBV DNA detection limit of the assay was 20 IU ml^−1^ or 108 copies per ml.

### HBsAg detection by immunofluorescence

CD2^−^ cells (non-T non-NK cells) were negatively isolated using CD2 microbeads (Miltenyi Biotec), according to manufacturer’s protocol. Subsequently, CD2^−^ cells were incubated for 2 h with or without recombinant HBsAg (10 μg ml^−1^; *adr* subtype) at 37 °C incubator and antigen uptake was stopped by two times cold wash in PBS. Cells were fixed and stained for HBsAg, using a two-step biotin–strepavidin staining protocol as previously described^32^. Cells were cytospinned onto Superfrost Plus slides (Thermo Scientific) using CYTO-TEK Cytocentrifuge (Sakura Finetek), sealed with ProLong Gold Antifade Reagent with DAPI (4',6-diamidino-2-phenylindole; Invitrogen) and HBsAg staining was visualized using TissueFAXS system (TissueGnostics). The exposure time for fluorescein isothiocyanate (FITC) filter (for HBsAg staining) on the microscope was adjusted based on the negative controls (healthy CB cells) and positive controls (healthy CB cells incubated with recombinant HBsAg), to minimize autofluorescence/background staining without compromising signal. The total number of HBsAg^+^ cells and the total number of DAPI-stained nuclei were manually counted in ten random high power fields ( × 20 magnification).

### Immunophenotyping

CB mononuclear cells were washed in PBS and stained with Live/Dead Fixable Dead Cell Stain. The cells were then washed in staining buffer (PBS, 1% BSA (Roche, Basel, Switzerland) and 0.1% sodium azide (Sigma-Aldrich)), stained for expressed cell surface molecules and analysed on a BD FACSAria or LSR II cytometer. T-bet staining for Th1 cells was performed using Human FoxP3 Buffer Set (BD), according to manufacturer’s protocol. Following overnight stimulation with phorbol myristate acetate (2 ng ml^−1^) and ionomycin (1 μg ml^−1^) in the presence of brefeldin A (2 μg ml^−1^), surface-stained cells were fixed and permeabilized (Cytofix/Cytoperm; BD) before being stained for produced cytokines. Cells were then washed in staining buffer with 0.1% saponin (Sigma-Aldrich) before acquisition on LSR II cytometer. Data were analysed using FACSDiva software (BD).

### Cell sorting/gating strategy

APCs were sorted/gated based on lineage markers (CD3/CD7/CD56/CD19/CD20) and HLA-DR expression: CD14^+^ monocytes (lineage^−^HLA-DR^+^CD14^+^CD16^low^) and pDCs (lineage^−^HLA-DR^+^CD14^−^CD16^−^CD11c^−^CD123^+^). T cells were gated based on CD3^+^ expression.

### Human immunology gene expression analysis

Cell lysates from 50,000 sorted CD14^+^ monocytes/pDCs were analysed using the preassembled nCounter GX Human Immunology Kit and the nCounter system (NanoString Technologies, Seattle, WA), according to the manufacturer’s instructions. Data analysis was performed as previously described^51^. Briefly, a cutoff of two times the mean of the negative controls supplied in the kit was used to discriminate against nonspecific probe binding (noise). Samples were then normalized based on the geometric means of both the positive controls supplied in the kit and the panel of housekeeping genes, as recommended by the manufacturer. The coefficient of variation (s.d. of the normalized counts across all samples/mean normalized counts across all samples, expressed as a percentage) of each gene was calculated and the mean coefficient of variation of the housekeeping genes was used as a cutoff to filter out genes that remain stable across all samples analysed.

### Clustering

Log_2_ normalized counts were used for clustering analysis. Data were normalized (mean centering of genes) and hierarchical clustering of genes was generated using Cluster 3.0 (similarity metric: Euclidean distance, clustering method: Average linkage) and visualized in TreeView.

### Mixed lymphocyte reaction

Pan T cells (10^5^ cells per well from a single healthy donor) were labelled with carboxyfluorescein succinimidyl ester (CFSE) and seeded in 96-well round-bottom plate with sorted CD14^+^ monocytes (ET=1). Pan T cells incubated with anti-CD3/CD28-coupled beads (Invitrogen, 1:1 bead per cell ratio) were used as positive control. After 7 days, cells were stained with anti-CD3-Horizon V450 (BD Biosciences) and acquired on a BD LSR-II flow cytometer. T-cell proliferation was assessed by CFSE dilution. Proliferation index was calculated using Flowjo software.

### Bacterial stimulation

CB mononuclear cells were seeded in 96-well U-bottom plate at 10^5^ cells per well in AIM-V media (Life Technologies) supplemented with 2% AB serum (Invitrogen). The number of monocytes was assumed at 10% of total cells. *P. aeruginosa*, *L. monocytogenes*, *UPEC*, *S. typhimurium* and *A. baumanii* were added to the culture at a multiplicity of infection 1 per monocyte and incubated overnight. Cell supernatant was collected and analysed for cytokine production with the multiplex assay (Luminex).

### Cytokine multiplex bead-based assay and ELISA

Cytokine concentrations in plasma samples and in supernatants of cultured cells were measured using MILLIPLEX MAP Human Cytokine/Chemokine Magnetic Bead Panel—Premixed 42 Plex (Millipore, Billerica, MA), according to manufacturer’s protocol. Analyte concentrations were determined by interpolation from a standard curve. ELISA of IL-12 and IL-23 (R&D Systems), as well as of IFN-β and IFN-λ (PBL Assay Science) were performed according to the manufacturer’s instructions.

### Recombinant cytokine stimulation

Healthy CB cells were seeded in 96-well U-bottom plate at 2 × 10^5^ cells per well in AIM-V media (Life Technologies) supplemented with 2% AB serum (Invitrogen). rhIL-12p40 (BD) and/or rhIFN-α2 (PBL, Piscataway, NJ) were added either alone or in different combinations of concentrations for 24 h and the activation of T cells and monocytes analysed using FACS (BD). The concentrations of rhIL-12p40 tested were 0.1–1,000 ng ml^−1^ and 0.0004–4 ng ml^−1^ for rhIFN-α2.

### Nuclei preparation and FISH analysis

Nuclei were prepared for FISH analysis by resuspending the cells in 7 ml of 0.075 mol l^−1^ KCl and incubating them at 37 °C water bath for 15 min. Two milliliters of 3:1 methanol:acetic acid was added to the cells, centrifuged and the pellet was resuspended and washed twice with 7 ml of methanol:acetic acid solution. Samples were stored at least overnight at −20 °C until slides were prepared. Nuclei were dropped onto methanol-cleaned slides and air dried overnight on a 56 °C hot plate.

Slides pretreatment was performed in the following order: 1 × PBS at room temperature for 5 min, pepsin/HCl solution at 37 °C for 5 min, 1 × PBS at room temperature for 5 min, 1% formaldehyde at room temperature for 10 min, 1 × PBS at room temperature for 5 min and dehydrated in successive washes of 70%, 80% and 100% ethanol at room temperature for 2 min each and allowed to air dry. Poseidon Chromosome X and Y Satellite Enumeration Probes were obtained from Kreatech (the Netherlands) and used according to manufacturer’s protocol. Nuclei were counterstained with DAPI solution. Post-hybridization washes were performed as per manufacturer’s instructions. Images were visualized and captured using the Isis Fluorescence Imaging System with the Nikon Eclipse 80i microscope. The number of maternal cells were manually counted in 20 random high-power fields and expressed as a percentage of total nuclei.

### High-throughput single-cell quantitative PCR

Cells were first sorted into 5 μl lysis buffer in 96-well plates (CellsDirect Resuspension and Lysis Buffer, Invitrogen) and snap-frozen with dry ice. Right before reverse transcription, samples were heated at 65 °C for 90 s and immediately snap chilled on ice for 5 min. Reaction buffer (1.4 μl) and 0.7 μl enzyme (Maxima First Strand cDNA Synthesis Kit, Thermo Scientific) were added to each sample and reverse transcription was performed with the following protocol: 10 min 25 °C, 15 min 50 °C, 5 min 85 °C. Sequence-specific pre-amplification was performed using TaqMan PreAmp Master Mix (Invitrogen, PN 4391128) by activating the enzyme at 95 °C for 10 min, denaturing at 96 °C for 5 s, then annealing and amplification at 60 °C for 4 min for 20 cycles. Unincorporated primers were inactivated by Exonuclease I by digesting at 37 °C for 30 min and inactivation at 80 °C for 15 min. The resulting complementary DNA was diluted fivefold in DNA Suspension Buffer (10 mM Tris, pH 8.0, 0.1 mM EDTA; TEKnova, PN T0221) before analysis with 2 × Sso Fast EvaGreen Supermix With Low ROX (Bio-Rad Laboratories, PN 172-5211) with nested primers in 48:48 Dynamic Arrays on a Biomark System (Fluidigm). Ct values were calculated from the system’s software (Biomark Real-time PCR analysis, Fluidigm). The list of primers used is shown in Supplementary Methods.

### Raw data treatment and visualization

All Raw Ct values were normalized to the assumed detection Ct level of 30, following recommendation from Fluidigm technical support. For visualization purposes, heatmaps were produced using custom scripts and ggplot package in R.

### Statistical analysis

The non-parametric Mann–Whitney *U*-test was used to determine the statistical significance of differences, unless otherwise stated, and *P*-values were denoted by *P<0.05, **P<0.01, ***P<0.001 and ****P<0.0001.

## Authors contributions

M.H. designed the study, performed the experiments, analysed the data and wrote the manuscript. E.S. collected the samples, designed the study, performed the experiments and analysed the data. D.L. and A.J.G. performed the experiments and analysed the data. S.F., B.A., S.U. and Y.-S.C. provided the samples and proofread the manuscript. E.G. designed the study, analysed the data and proofread the manuscript. A.B. designed the study, analysed the data and wrote the manuscript.

## Additional information

**Accession codes**: The Nanostring data reported in the paper have been deposited in the NCBI GEO database under accession number GSE65389.

**How to cite this article:** Hong, M. *et al.* Trained immunity in newborn infants of HBV-infected mothers. *Nat. Commun.* 6:6588 doi: 10.1038/ncomms7588 (2015).

## Supplementary Material

Supplementary Information Supplementary Figures 1-8, Supplementary Tables 1-2 and Supplementary Methods
