# Supplementary material for: Trained immunity in newborn infants of HBV-infected mothers
Source: Nat Commun. 2015 Mar 25;6:6588. doi: 10.1038/ncomms7588 (PMC4389241; doi:10.1038/ncomms7588)
Supplement: Supplementary Information — Supplementary Figures 1-8, Supplementary Tables 1-2 and Supplementary Methods [file ncomms7588-s1.pdf]

Supplementary Figure 1

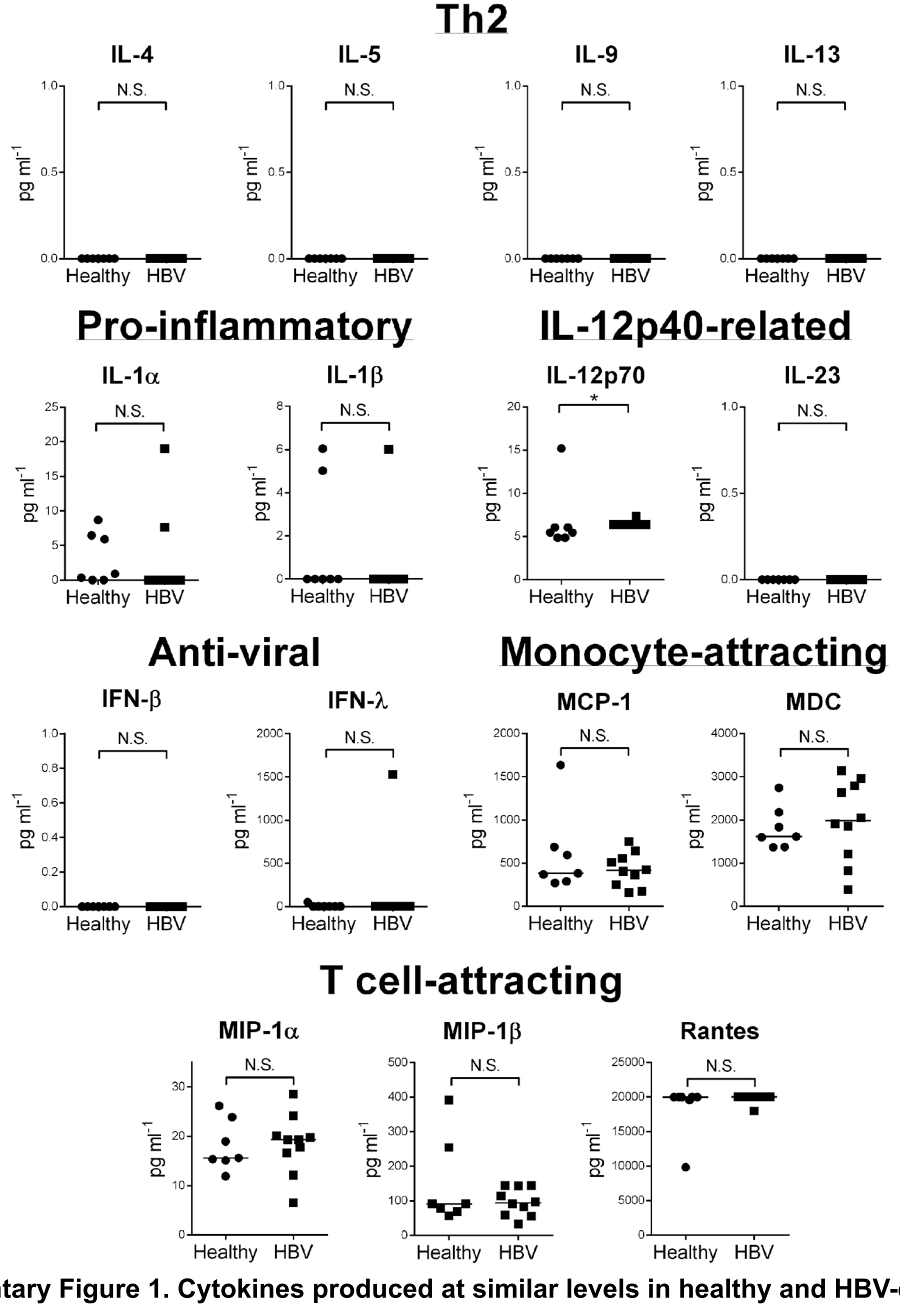

Supplementary Figure 1. Cytokines produced at similar levels in healthy and HBV-exposed cord blood of Asian HBV^+ mothers. The production (in pg ml^{-1}) of Th2 cytokines (IL-4, IL-5, IL-9, IL-13), pro-inflammatory cytokines (IL-1α, IL-1β), IL-12p40-related cytokines (IL-12p70, IL-23), anti-viral cytokines (IFN-β, IFN-λ), monocyte-attracting (MCP-1, MDC) and T cell-attracting chemokines (MIP-1 α, MIP-1β, Rantes) in the CB plasma of healthy (n=7) and HBV^+ (n=10) Asian mothers were determined using luminex or ELISA. P-values were calculated using Mann-Whitney U test, one-tailed. N.S. denotes non-significant.

Supplementary Figure 2

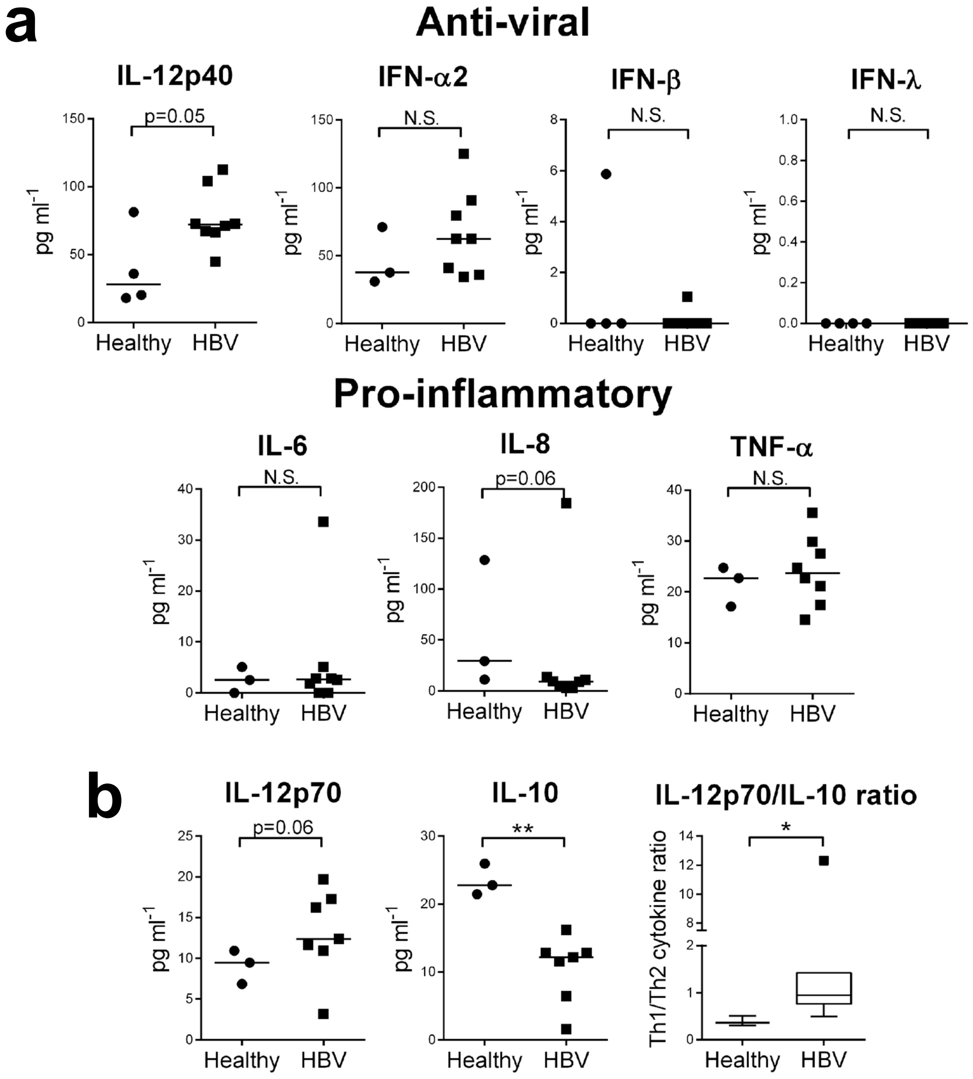

Supplementary Figure 2. High IL-12p40, low IL-10, and increased Th1/Th2 cytokine ratio in cord blood of Caucasian HBV⁺ mothers than controls. (**a**) The production (in pg ml^{-1}) of anti-viral cytokines (IL-12p40, IFN-α2, IFN-β, IFN-λ), pro-inflammatory cytokines (IL-6, IL-8, TNF-α), and (**b**) Th1 (IL-12p70), Th2 (IL-10) cytokine as well as the ratio of Th1/Th2 cytokine in healthy (n=4) and HBV-exposed (n=8) CB plasma from Caucasian HBV^+ mothers. Statistical difference was calculated using Mann-Whitney U test, one-tailed. *, ** represent P<0.05, P<0.01 respectively. N.S. denotes non-significant.

Supplementary Figure 3

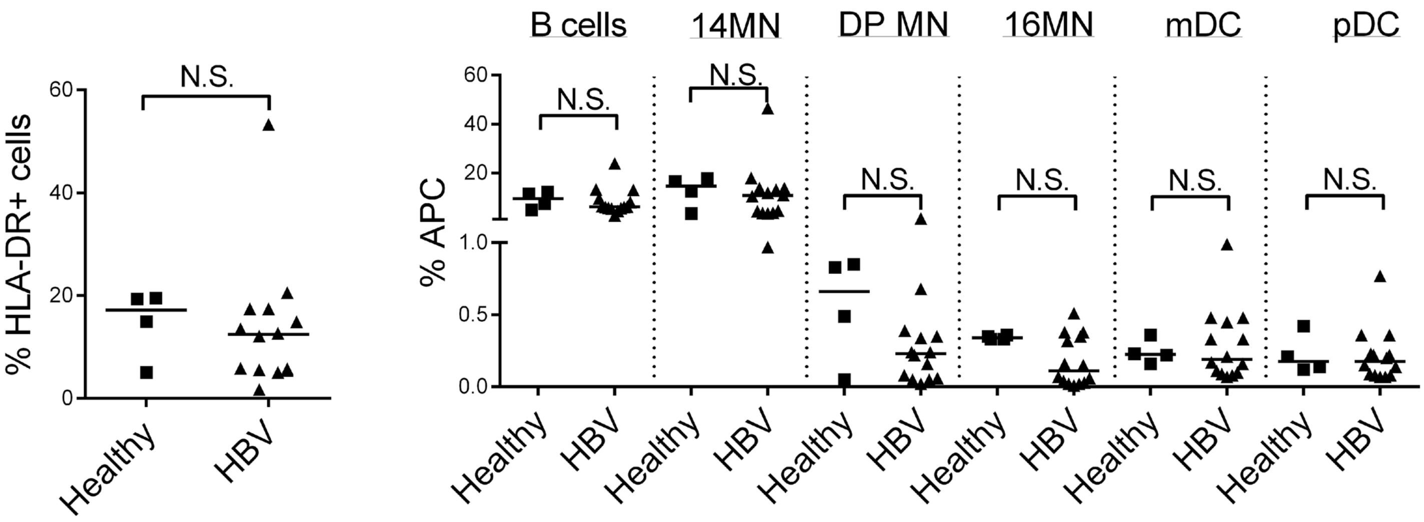

Supplementary Figure 3. HBV exposure does not affect the frequencies of antigen presenting cells in cord blood. Graphs show the percentages of total antigen-presenting cells, or APCs (i.e. HLA-DR^{+} cells) and the different APC subsets in cord blood from healthy (n=4) and HBV^{+} (n=14) mothers. Horizontal lines represent the median. Statistical difference was calculated using Mann-Whitney U test, one-tailed. N.S. denotes non-significant.

Supplementary Figure 4

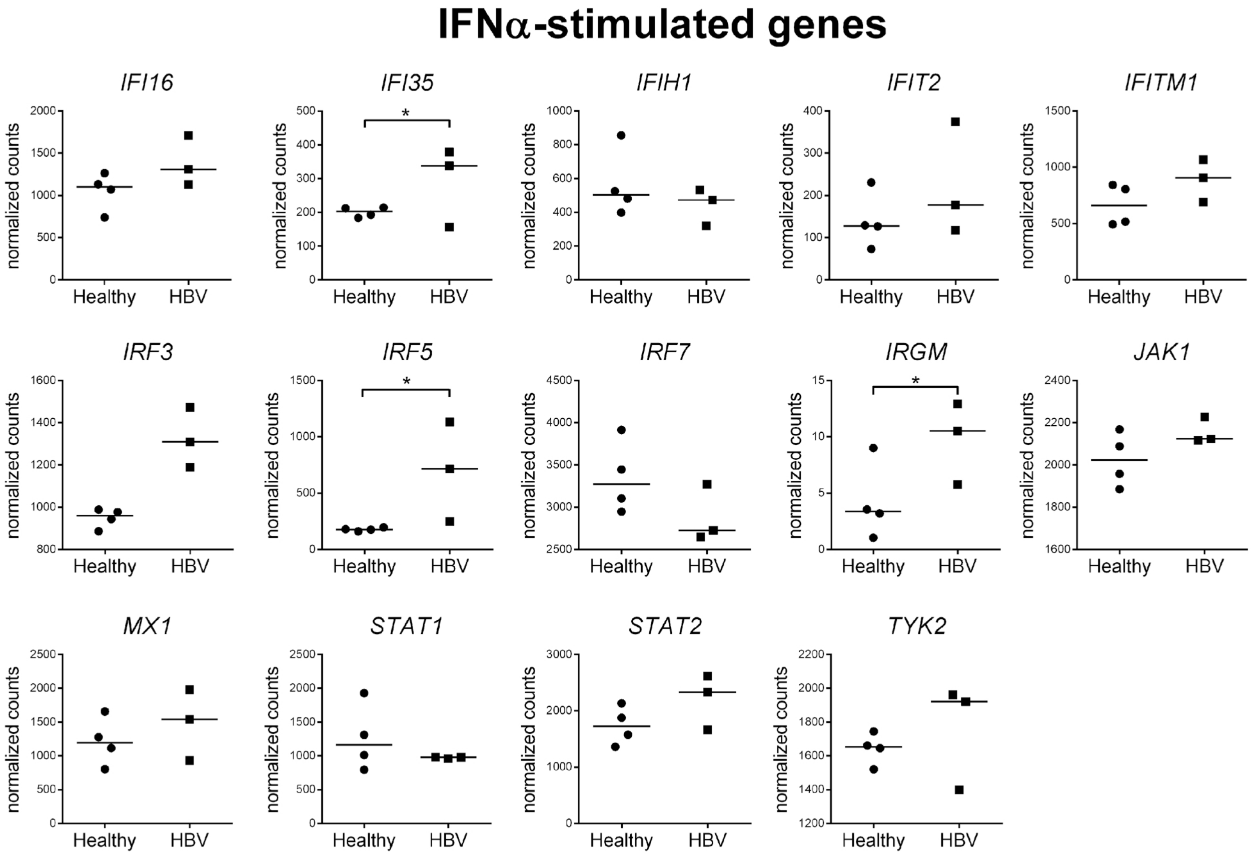

Supplementary Figure 4. Higher expression of interferon stimulated genes in HBV-exposed CB monocytes than controls. The mRNA expression (in Nanostring counts) of interferon-stimulated genes, or ISGs, in healthy (n=4) and HBV-exposed (n=3) CB monocytes. Statistical difference was performed using Mann-Whitney U-test, one-tailed. * denotes P<0.05.

Supplementary Figure 5

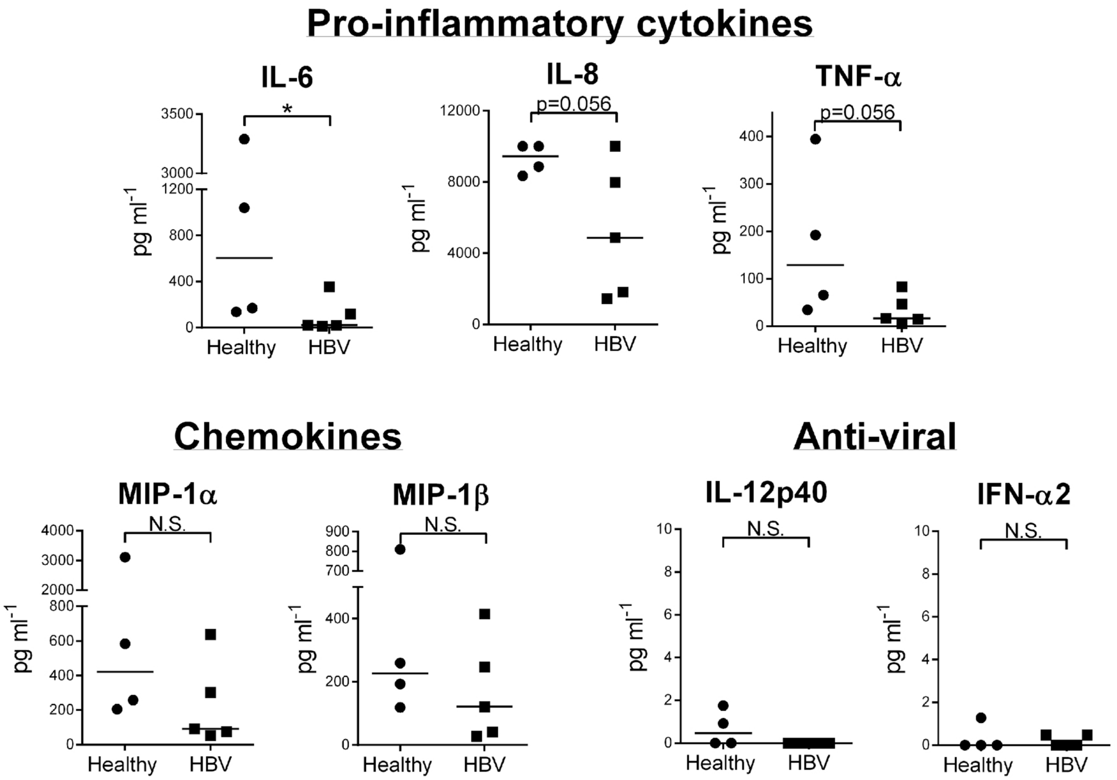

Supplementary Figure 5. HBV-exposed CB monocytes produce lower levels of pro-inflammatory cytokines than controls. The production (in pg ml^{-1}) of pro-inflammatory cytokines (IL-6, IL-8, TNF-α), chemokines (MIP-1α, MIP-1β), and anti-viral cytokines (IL-12p40, IFN-α2) in the supernatant of sorted healthy (n=4) and HBV-exposed (n=5) CB monocytes after overnight culture was determined using luminex. P-values were calculated using Mann-Whitney U test, one-tailed. * denotes $P<0.05$. N.S. indicates non-significant.

Supplementary Figure 6

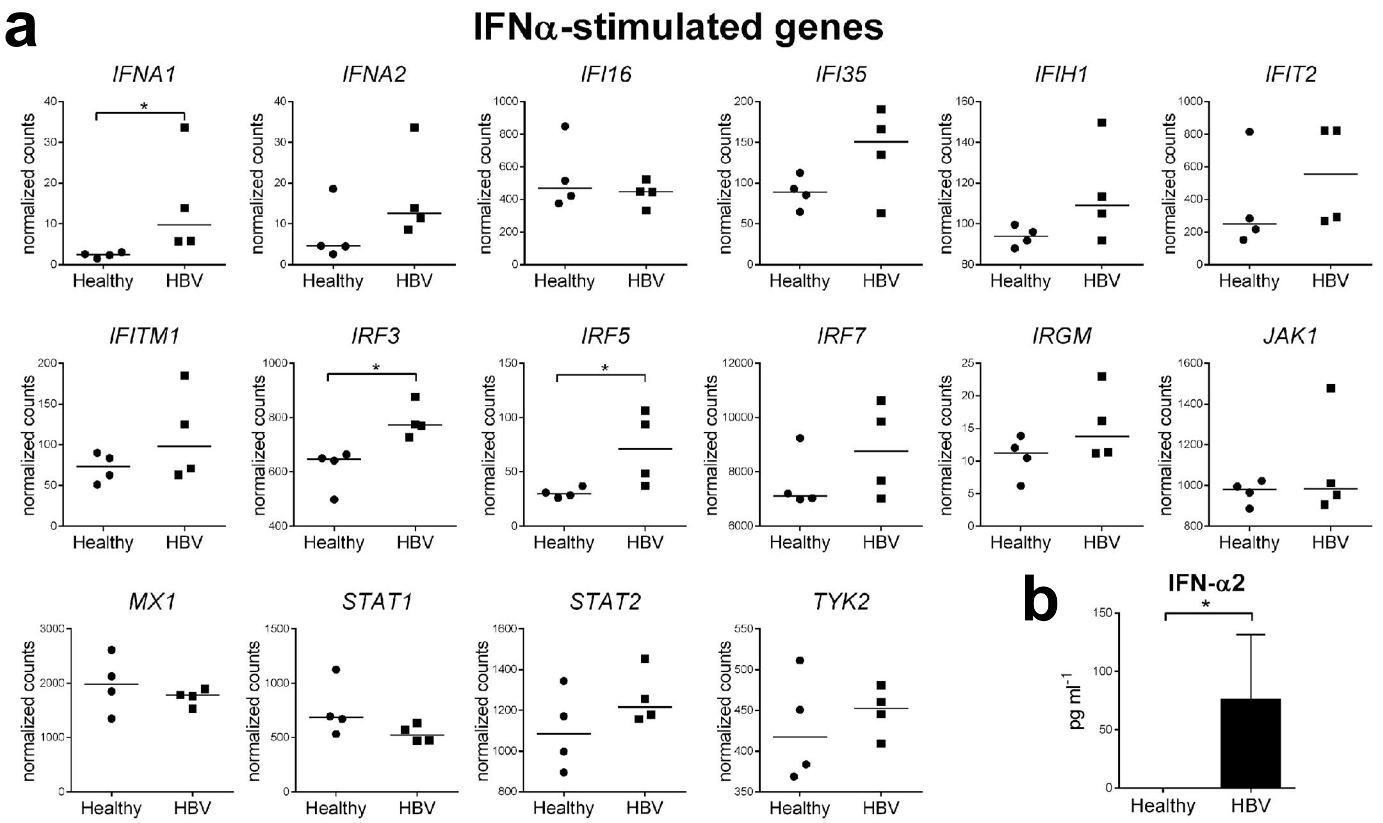

Supplementary Figure 6. Increased expression of interferon stimulated genes and higher production of IFN-α2 in HBV-exposed CB plasmacytoid DC. (**a**) The mRNA expression (in Nanostring counts) of ISGs in healthy (n=4) and HBV-exposed (n=4) CB pDCs. (**b**) The production of IFN-α2 (in pg ml^{-1}) in the supernatant of pDCs stimulated for 18h with TLR9 agonist (CpG ODN2216; 5μM). Statistical difference was performed using Mann-Whitney U-test, one-tailed. * denotes $P<0.05$.

Supplementary Figure 7

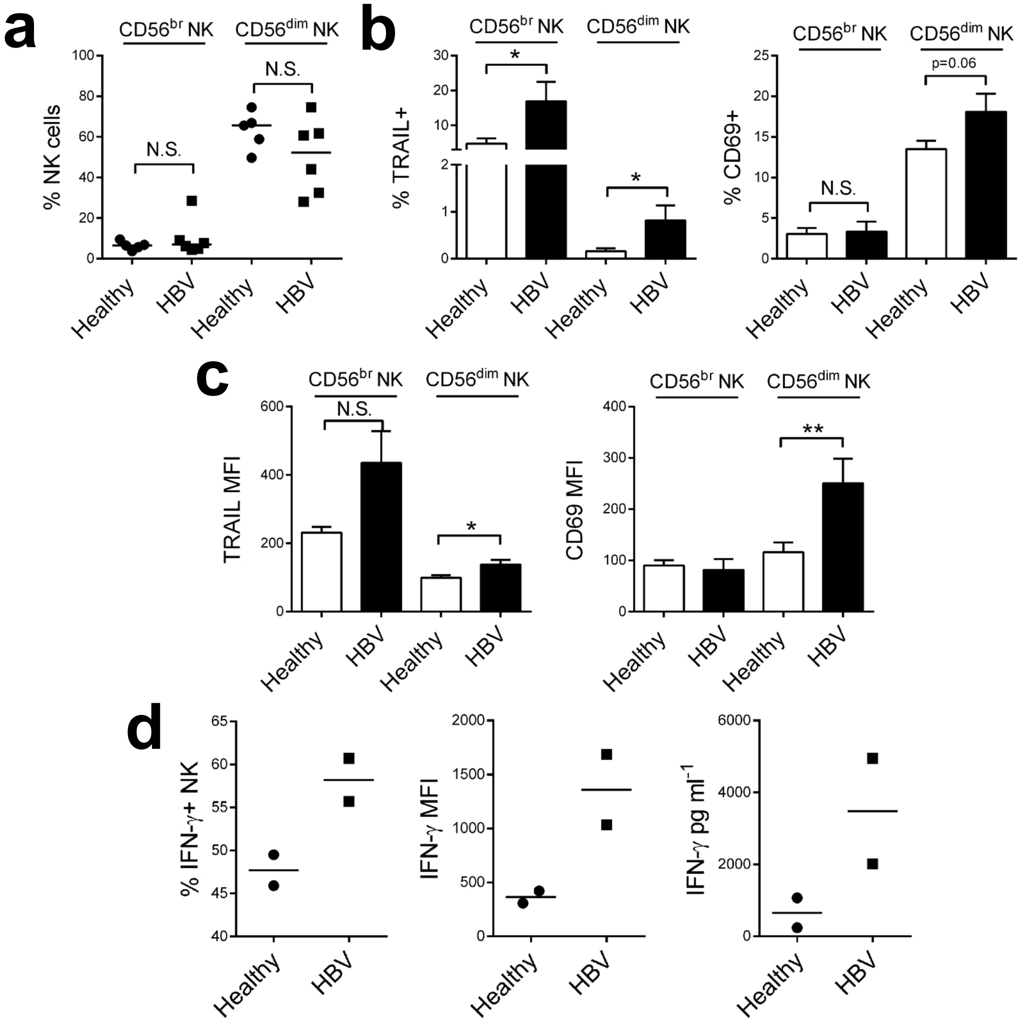

Supplementary Figure 7. HBV-exposed CB NK cells were similar in frequency but display an activated profile than controls. (**a**) No difference in the frequencies of CD56^{br} and CD56^{dim} NK cells in healthy (n=5) and HBV-exposed (n=6) CB analyzed by flow cytometry (mean±SEM in percentages; CD56^{br} NK, healthy, 6.5±0.9, HBV, 10.2±3.7l; CD56^{dim} NK, healthy, 63.1±4.2, HBV, 50.2±7.5). (**b**) Higher frequency of TRAIL-expressing CD56^{br} and CD56^{dim} NK cells (mean±SEM in percentages; CD56^{br} NK, healthy, 4.7±1.5, HBV, 16.9±5.6; CD56^{dim} NK, healthy, 0.2±-0.1, HBV, 0.8±0.3), and a trend for increased frequency of CD69-expressing CD56^{dim} NK cells in HBV-exposed CB compared to controls (mean±SEM in percentages; CD56^{dim}, healthy, 13.5±1, HBV, 18.1±1.2). (**c**) Higher TRAIL and CD69 MFI on CD56^{dim} NK cells in HBV-exposed CB than controls (MFI; TRAIL, healthy, 99.4±7.2, HBV, 138±14; CD69, healthy, 116.2±19.2, HBV, 250.7±48). (**d**) Higher frequency IFN-γ^{+} NK cells and increased IFN-γ MFI expression by ICS staining, and elevated production of IFN-γ in the supernatant by luminex analysis in HBV-exposed CB NK cells (n=2) compared to healthy controls (n=2) after over-night stimulation with recombinant human IL-12p70 and IL-18 (50ng ml^{-1} each). Statistical significance was performed using Mann-Whitney U test, one-tailed. *, ** denote P<0.05, P<0.01 respectively. N.S. represents non-significant.

Supplementary Figure 8

HBV status of mother	HLA-A2	HBV pentamers	No. of CB tested	No. of CB tested negative for HBV pentamers ex vivo
HBsAg negative	positive	HBV core 18-27	4	4
HBsAg positive	positive	HBV env 183-191	6	6
HBsAg negative	negative	HBV pol 573-581	1	1

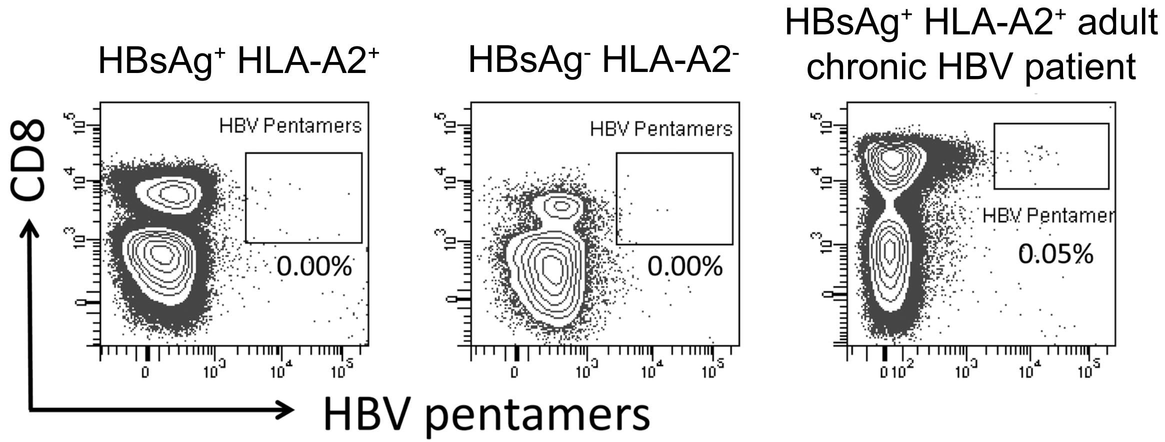

Supplementary Figure 8. HBV-specific T cells were not detected *ex vivo* in cord blood of HBV+ mothers. Three different pentamers, complex of HLA-A2 and HBV core 18-27 peptide or envelope 183-191 or polymerase 573-581 were used. A representative dot plot of pentamer staining for an adult chronic HBV patient is shown.

Supplementary Table 1. Clinical and demographic characteristics of study groups and the list of experiments performed in each sample

	Cord blood characteristics					Maternal characteristics					Cord blood experiments											
	S/N	Sample ID	Clinical Diagnostic	Gender	Plasma[a]	HBV DNA (log copies ml^{-1})	HBsAg status	HBeAg status	HBV genotype	Anti-viral treatment during pregnancy	Plasma cytokine	Monocyte nanostring	T cell cytokine ICS	Bacteria stimulation	Plasma HBV DNA qPCR (log copies ml^{-1})[b]	CBMC HBV DNA qPCR (log copies ml^{-1})[b]	pDC analysis	NK analysis	Maternal cell FISH analysis	Maternal cell single cell PCR	HBsAg staining	In vitro rIFN-a2 + rIL-12p40 stimulation
Singapore	1	CB#1	Healthy	M	√	NIL	NIL	NIL	NIL	NIL	√								√		√	
	2	CB#2	Healthy	F	√	NIL	NIL	NIL	NIL	NIL	√	√	√				√				√	
	3	CB#3	Healthy	M	√	NIL	NIL	NIL	NIL	NIL	√	√	√	√	√	√ (ND)	√	√	√		√	√
	4	CB#4	Healthy	?	√	NIL	NIL	NIL	NIL	NIL	√	√	√	√	√		√	√			√	
	5	CB#5	Healthy	?	√	NIL	NIL	NIL	NIL	NIL	√	√	√		√	√ (ND)	√	√			√	√
	6	CB#6	Healthy	?	√	NIL	NIL	NIL	NIL	NIL	√		√		√			√			√	√
	7	CB#7	Healthy	?	√	NIL	NIL	NIL	NIL	NIL	√		√		√			√			√	
	1	CB#8	HBV	M	√		+	-	B/C	-					√							
	2	CB#9	HBV	M	√		+	-	B/C	-	√		√		√	√ (ND)			√		√	
	3	CB#10	HBV	M	√		+	+	B/C	-	√		√		√ (5.55)	√ (ND)					√	
	4	CB#11	HBV	F	√		+	-	B/C	-	√		√	√	√						√	
	5	CB#12	HBV	M	√	<2	+	-	B/C	-	√		√		√							
	6	CB#13	HBV	F			+	-	B/C	-												
	7	CB#14	HBV	M			+	-	B/C	-			√			√ (ND)						
	8	CB#15	HBV	F	√		+	+	B/C	-	√		√	√	√						√	
	9	CB#16	HBV	F			+	+	B/C	-			√									
	10	CB#17	HBV	F		4.58	+	-	B/C	-							√	√				
	11	CB#18	HBV	F	√		+	-	B/C	-	√				√			√				
	12	CB#19	HBV	F	√		+	-	B/C	-	√	√	√		√		√				√	
	13	CB#20	HBV	M	√		+	+	B/C	-	√		√		√				√	√		
	14	CB#21	HBV	F	√		+	+	B/C	-	√	√	√		√ (3.74)		√				√	
	15	CB#22	HBV	F	√		+	-	B/C	-			√		√			√				
	16	CB#23	HBV	M	√		+	-	B/C	-	√				√			√				
	17	CB#24	HBV	F	√		+	-	B/C	-					√							
	18	CB#25	HBV	M	√		+	-	B/C	-		√			√		√					
	19	CB#26	HBV	M			+	+	B/C	-								√				
	20	CB#27	HBV	F	√		+	-	B/C	-					√							
Italy	1	CB#28	Healthy	?	√	NIL	NIL	NIL	NIL	NIL	√			√								
	2	CB#29	Healthy	F	√	NIL	NIL	NIL	NIL	NIL	√			√								
	3	CB#30	Healthy	F	√	NIL	NIL	NIL	NIL	NIL	√			√								
	4	CB#31	Healthy	?	√	NIL	NIL	NIL	NIL	NIL	√											
	1	CB#32	HBV	M	√	3	+	+		-	√			√								
	2	CB#33	HBV	M	√	2.06	+	+		-	√											
	3	CB#34	HBV	F	√	2.64	+	-		-	√			√								
	4	CB#35	HBV	M	√	<2	+	-		-	√											
	5	CB#36	HBV	M	√	5.31	+	-		-	√											
	6	CB#37	HBV	F	√	2.69	+	-		-	√											
	7	CB#40	HBV	M	√	9.27	+	+		-	√			√								
	8	CB#41	HBV	?	√	<2	+	-		-	√											

[a] Cord blood plasma was collected after Ficoll-Hypaque gradient separation of the umbilical cord blood from neonates of healthy (n=7), $HBeAg^-$ (n=11), and $HBeAg^+$ (n=4) mothers from Singapore cohort, and from neonates of healthy (n=4), $HBeAg^-$ (n=5), and $HBeAg^+$ (n=3) mothers from Caucasian cohort.

[b] Quantification of HBV DNA was performed using Qiagen artus HBV RG PCR Kit and quantitative real-time PCR. ND denotes non-detectable.

Supplementary Table 2. Two-way ANOVA table with Bonferroni's post-test

a Two-way ANOVA of MHC class II processing and presentation gene expression

Source of variation	% of total variation	P value
Interaction	2.541	N.S.
Patient group	1.274	<0.05
Gene expression	85.68	<0.0001

Bonferroni's Multiple Comparison Test of MHC class II processing and presentation gene expression

Gene	95% CI of difference	P value
HLA-DPA1	-11925 to 10944	N.S.
HLA-DPB1	-12029 to 10840	N.S.
HLA-DQA1	-12184 to 10686	N.S.
HLA-DQB1	-12279 to 10590	N.S.
HLA-DRA	-16880 to 5989	N.S.
HLA-DRB1	-25289 to -2420	<0.01
HLA-DRB3	-13389 to 9480	N.S.
CIITA	-12173 to 10696	N.S.

b Two-way ANOVA of Complement components gene expression

Source of variation	% of total variation	P value
Interaction	6.973	N.S.
Patient group	14.6	<0.0001
Gene expression	59.19	<0.0001

Bonferroni's Multiple Comparison Test of Complement components gene expression

Gene	95% CI of difference	P value
C1QA	-39.80 to 21.51	N.S.
C1QB	-73.54 to -12.23	<0.01
C1S	-37.55 to 23.76	N.S.
C3	-41.34 to 19.98	N.S.
C4B	-52.74 to 8.576	N.S.
C5	-42.87 to 18.44	N.S.
CFB	-73.04 to -11.72	<0.01

c Two-way ANOVA of Chemokines & receptors gene expression

Source of variation	% of total variation	P value
Interaction	7.202	N.S.
Patient group	5.223	0.06
Gene expression	23.1	0.06

Bonferroni's Multiple Comparison Test of Chemokines & receptors gene expression

Gene	95% CI of difference	P value
CCL3	-8961 to 35812	N.S.
CCL4	-11711 to 33062	N.S.
CCR2	-22400 to 22373	N.S.
CCR5	-22390 to 22383	N.S.
CXCL1	-13620 to 31153	N.S.
CXCL2	-10529 to 34244	N.S.
CXCR4	-23050 to 21723	N.S.
CCL15	-22406 to 22367	N.S.
CXCL13	-22396 to 22377	N.S.

d Two-way ANOVA of Cytokines & receptors gene expression

Source of variation	% of total variation	P value
Interaction	21.86	<0.0001
Patient group	5.593	<0.0001
Gene expression	67.28	<0.0001

Bonferroni's Multiple Comparison Test of Cytokines & receptors gene expression

Gene	95% CI of difference	P value
IFNA2	-81.15 to 68.14	N.S.
IFNG	-77.07 to 72.22	N.S.
IL10	-37.68 to 111.6	N.S.
IL12A	-81.97 to 67.32	N.S.
IL12B	-83.07 to 66.21	N.S.
IL15	-403.6 to -254.3	<0.0001
TNFRSF17	-85.06 to 64.22	N.S.
TNFSF11	-86.25 to 63.04	N.S.
TNFSF8	-342.8 to -193.5	<0.0001
IL12RB1	-83.48 to 65.81	N.S.
LTA	-98.55 to 50.74	N.S.

e Two-way ANOVA of Signaling molecules gene expression

Source of variation	% of total variation	P value
Interaction	3.196	<0.01
Patient group	5.82	<0.0001
Gene expression	84.46	<0.0001

Bonferroni's Multiple Comparison Test of Signaling molecules gene expression

Gene	95% CI of difference	P value
IKBAP	-405.3 to 289.8	N.S.
IKBKB	-686.9 to 8.258	N.S.
IKBKG	-513.9 to 181.3	N.S.
IRF3	-531.3 to 163.9	N.S.
IRF5	-962.1 to -266.9	<0.0001
MAP4K1	-458.9 to 236.3	N.S.
MAP4K2	-442.0 to 253.1	N.S.
MAP4K4	-883.1 to -187.9	<0.001
STAT2	-777.4 to -82.23	<0.01
STAT5A	-688.9 to 6.251	N.S.
IRAK1	-435.7 to 259.5	N.S.
IRAK4	-394.5 to 300.6	N.S.
TRAF6	-521.0 to 174.2	N.S.

f Two-way ANOVA of Pro-inflammatory cytokines gene expression

Source of variation	% of total variation	P value
Interaction	17.63	<0.05
Patient group	5.871	<0.05
Gene expression	38.69	<0.001

Bonferroni's Multiple Comparison Test of Pro-inflammatory cytokines gene expression

Gene	95% CI of difference	P value
IL1A	-89090 to 89882	N.S.
IL1B	-19598 to 159375	N.S.
IL1R2	-89085 to 89888	N.S.
IL1RAP	-89148 to 89825	N.S.
IL1RN	-82816 to 96156	N.S.
IL6	-88807 to 90165	N.S.
IL8	48189 to 227161	<0.001
TNF	-87012 to 91960	N.S.

SUPPLEMENTARY METHODS

List of primers used for single-cell PCR:

**Primer Name**	**Primer Sequence**
TTY1_F	ACCCAGGACAAAGGAGGAGT
TTY1_R	GAGCAGACATCTCCCAGGTT
TTY1_nested_F	TGATGTGAAGCACGTGTTCA
XIST_F	TTGAAGATACCACGCTGCAT
XIST_R	AGCTTGGCCAGATTCTCAAA
XIST_nested_F	TGAGCATGTGAGACCTGAGG
XKRY_F	CAGGGAAGAATGCCAGAGTC
XKRY_R	GTAGGTGCCATTCACTGCAA
XKRY_nested_F	TAATGGCAGCAAAGTCGTTG
